# Inferring modules of functionally interacting proteins using the Bond Energy Algorithm

**DOI:** 10.1186/1471-2105-9-285

**Published:** 2008-06-17

**Authors:** Ryosuke LA Watanabe, Enrique Morett, Edgar E Vallejo

**Affiliations:** 1 ITESM Campus Estado de México, Carretera Lago de Guadalupe km 3.5, Atizapán de Zaragoza, 52926, México; 2 Departamento de Ingeniería Celular y Biocatálisis, Instituto de Biotecnología UNAM, Av. Universidad 2001, Cuernavaca, 62210, México

## Abstract

**Background:**

Non-homology based methods such as phylogenetic profiles are effective for predicting functional relationships between proteins with no considerable sequence or structure similarity. Those methods rely heavily on traditional similarity metrics defined on pairs of phylogenetic patterns. Proteins do not exclusively interact in pairs as the final biological function of a protein in the cellular context is often hold by a group of proteins. In order to accurately infer modules of functionally interacting proteins, the consideration of not only direct but also indirect relationships is required.

In this paper, we used the Bond Energy Algorithm (*BEA*) to predict functionally related groups of proteins. With *BEA *we create clusters of phylogenetic profiles based on the associations of the surrounding elements of the analyzed data using a metric that considers linked relationships among elements in the data set.

**Results:**

Using phylogenetic profiles obtained from the Cluster of Orthologous Groups of Proteins (*COG*) database, we conducted a series of clustering experiments using *BEA *to predict (upper level) relationships between profiles. We evaluated our results by comparing with *COG's *functional categories, And even more, with the experimentally determined functional relationships between proteins provided by the *DIP *and *ECOCYC *databases. Our results demonstrate that *BEA *is capable of predicting meaningful modules of functionally related proteins. *BEA *outperforms traditionally used clustering methods, such as *k*-means and hierarchical clustering by predicting functional relationships between proteins with higher accuracy.

**Conclusion:**

This study shows that the linked relationships of phylogenetic profiles obtained by *BEA *is useful for detecting functional associations between profiles and extending functional modules not found by traditional methods. *BEA *is capable of detecting relationship among phylogenetic patterns by linking them through a common element shared in a group. Additionally, we discuss how the proposed method may become more powerful if other criteria to classify different levels of protein functional interactions, as gene neighborhood or protein fusion information, is provided.

## Background

The development of automated methods for inferring functional association of proteins from sequence and genomic data is becoming an increasingly important area of investigation in bioinformatics and computational biology. In effect, the determination of unknown gene interactions in functional pathways and perhaps, their association with diseases, relies crucially on sound computational algorithms capable of producing meaningful predictions.

The homology-based methods are useful to assign function to proteins by establishing sequence similarity of the underlying sequences with others with previously assigned function [1]. There is a variety of those homology-based methods that use specific aspects of similarity between protein attributes, such as similarity between complete sequences [2-4], presence of motifs and functional blocks [5], specific spatial positions of functional residues [6,7] or combinations of the aforementioned [8].

However, when similarity of the underlined sequences is not sufficiently significant, alternative approaches have been considered. Several non homology-based methods have been developed to predict functional relationships between proteins [9,10], using additional sources of genomic information [11-13]. These methods have been called application-based in context, and they include phylogenetic profiles, protein fusions (Rosetta Stone), gene coexpresion, and neighborhood conservation [10]. It has been demonstrated that functional relationships, functional modules, molecular networks and genotype-phenotype relationships can be accurately predicted using these methods [10].

Among modern post-genomic approaches developed in recent years, those based on the correlated presence and absence of genes (i. e. phylogenetic profiles) among a collection of organisms have proven to be particularly effective [14,15]. Theoretically, with the increasing availability of complete genome sequences from more organisms, these methods hold the promise of increasing efficacy. Particularly, phylogenetic profiles have been successfully used for assigning protein function, for localizing proteins in cells, for reconstructing metabolic pathways, and for phylogenetic studies, among other applications [11,16-18].

Predictions obtained from phylogenetic profiles depend critically on the employed clustering method. Most clustering algorithms used to date are based on the calculation of Euclidean and Hamming distances between pair of elements [19], which means that the clustering is directed by the intrinsic properties of these patterns and no additional information is often considered, albeit there are few exceptions [17]. For example, other studies have employed a variety of metrics such as intergenic distance [18] and kernels [11]. It is known that proteins do not only interact by pairs [20]. For example, in the case of metabolic pathways, a biologically meaningful function is normally performed by a group of proteins. Also, our previous work demonstrated that often proteins can have functional analogs with no sequence similarity that can displace each other [15]. Thus, in order to predict a link, it is necessary to employ a method capable of considering not only a direct but also an indirect relationship created by the association with a third party element. The Bond Energy Algorithm (*BEA*) is a widely used methodology to create vertical fragmentation of distributed databases. This algorithm creates a collection of clusters based on the relationships of the surrounding elements of the analyzed data in a particular cluster using an non-trivial Attribute Affinity measure, which is a weight matrix denoting the strength of the relationship among all the elements in the data set. We would like to posit here that non obvious relationships can be predicted by this method when an apparently non related element is linked to another one by a third element [21,22]. *BEA *uses a two step approach to clustering: the first step is the ordering algorithm, while the second step is the grouping algorithm.

Despite its clustering capabilities, *BEA *has only recently been used in bioinformatics studies [22]. We propose a new method for clustering phylogenetic profiles, consisting in applying *BEA*, to predict functional associations of profiles and to detect displacements of functionally equivalent analog proteins. Here we show that *BEA *can detect functional associations between profiles not detected by conventional methods. A major goal of this work is to explore the extent in which the prediction of protein functional interactions can be accurately inferred from protein phylogenetic profiles. Therefore, we have focused on improving the clustering of these patterns by using implicit information not considered by most clustering methods. It is also possible to consider explicit domain knowledge in order to improve results in all clustering algorithms (e.g. an underlying metric incorporating knowledge constraints). Similarly, a consequence of our study is to find whether or not the implicit information derived from phylogenetic patterns is useful for inferring functional interactions without the resort of additional domain knowledge.

## Results and Discussion

### Experiments

The first version of the *COG *database provides a collection of 3307phylogenetic profiles [23,24]. Using this information we conducted a series of computational simulations with *BEA *and compared our results with those produced by other clustering methods, such as *k*-means, Hierarchical Clustering and Partition Around Medoids (*PAM*).

A predefined collection of input parameters is required by most clustering algorithms. The number of clusters, the initial position of cluster centers, and the distance metric, are typically provided as input to the clustering algorithm. Appropriate values for parameters are often determined empirically, especially, when there is little information on the underlying structure of the data set. The definition of these parameters is known to be critical for obtaining meaningful clustering results [25].

For instance, in the *k*-means algorithm we used a random positioning of 18 centers for the clusters. The determination of this number of centers was based on the number of different functional categories described in the *COG *database. Recall that *COG *phlogenetic profiles and *COG *functional categories are independent assessments. The selected metric was the Hamming distance due to the fact that it is a suitable measure for binary data. The accuracy of the algorithm was calculated as the average performance of 10 simulations of the algorithm based on the ten-fold validation method [26,27]. The Matlab package [28] was employed for this experiment.

Results produced by *k*-means are typically sensitive to initial conditions. For example, a particular partition of points in which any single point is moved to a different cluster increases the total sum of distances. This problem can be approached by an appropriate choice of starting points. Specifically, we employed a version of the *k*-means algorithm that uses a two-phase iterative algorithm to minimize the sum of point-to-centroid distances, summed over all *k *clusters [25].

The first phase use "batch" updates, in which each iteration consists of re-assigning points to their nearest cluster centroid, all at once, followed by recalculation of cluster centroid. This phase may be viewed as providing a fast but potentially only approximate solution and as a starting point for the second phase. The second phase use "online" updates, in which points are individually reassigned in the space in order to reduce the sum of distances; cluster centroids are recomputed after each reassignment. Each iteration during this second phase consists of stepwise pass though all the points to assign the new centroids. For the Hierarchical Clustering experiments, we used the R package statistical toolkit (AGNES). AGNES is fully described in [25,29]. Compared to other agglomerative clustering methods such as "hclus", "agnes" yields the agglomerative coefficient which measures the amount of clustering structure found.

The AGNES algorithm constructs a hierarchy of clusters. Initially each observation is a small cluster by itself. Clusters are merged until only one large cluster remains which contains all the observations. At each stage of the algorithm the two nearest clusters are combined to form a single larger cluster. We used the average method in which the distance between two clusters is the average of the dissimilarities between the points in one cluster and the points in the other. The obtained agglomerative coefficient of 0.7788952 provided the basis to cut the branch at the coefficient value. The chosen metric was again the Hamming distance. As mentioned above, we repeated 10 cycles and used the average result of them.

For the experiments with *PAM *we also used the R package statistical toolkit. 18 clusters was provided as input parameter to the algorithm as for "*k*-means". *PAM *is a more robust version of *k*-means since it additionally takes a dissimilarity matrix as input. *PAM *algorithm is based on the search for *k *representative objects or medoids among the observations of the data set. These observations should represent the structure of the data. After finding a set of *k *medoids, *k *clusters are constructed by assigning each observation to the nearest medoid. The goal is to find *k *representative objects which minimize the sum of the dissimilarities of the observations to their closest representative object. By default, when medoids are not specified, the algorithm first looks for a good initial set of medoids (this is called the build phase). Then, it finds a local minimum for the objective function, that is, a solution reached until there is not a single switch of an element within a medoid that decreases the objective function (this is called the swap phase). When the medoids are specified, their order is not critical, in general, the algorithms have been designed to be independ of the order of the observations. The metric was set to Manhattan, as before. We hold 10 cycles and took the average result for a better accuracy.

In contrast, for the experiments with *BEA*, the number of clusters was automatically created by the cutting method in conjunction with a grouping step based on the "guilty by association" principle. The Hamming was also used for the experiments with *BEA *and we hold 10 cycles, as before.

A more detailed description of *BEA *is presented in the Methods section.

## Results

In this study we used *BEA *to classify phylogenetic profiles obtained from the *COG *database and conduct a series of experiments with experimental and theoretical data such as the *COG *Functional Categories, *DIP*, and *ECOCYC *databases. These tests provide a measurement of the biological significance of our results. We have chosen the aforementioned databases to evaluate our clustering algorithm as they provide a large collection of experimentally confirmed protein-interaction pairs. In addition, these databases are based on COGs, which provide uniformity with respect to the data set used in this study. It would also be interesting to evaluate our predictions with metabolic pathway and/or functional categories which likely will produce similar results. See Table 1.

**Table 1:** Resulting classification for all methods

Code	COGs	PREDICTED BEA	PREDICTED HIERARCHICAL	PREDICTED K-MEANS	PREDICTED PAM
Information storage and processing					
J	217	213	321	544	754
K	132	122	4	0	0
L	184	181	39	0	0
Cellular processes					
D	32	32	0	0	0
O	110	104	26	0	0
M	155	153	140	84	0
N	133	130	46	81	100
P	160	149	10	0	0
T	97	83	0	0	0
Metabolism					
C	224	220	65	24	0
G	171	164	17	197	62
E	233	226	584	260	312
F	85	83	18	0	0
H	154	141	49	0	0
I	75	72	0	0	0
Q	62	55	0	0	0
Poorly characterized					
R	449	431	113	250	0
S	750	748	1875	1867	2079

Classification for COG Functional Categories (One classification for multiple classification COGs).

### Validations with COG functional categories

For the first testing, we used the functional categories established by the *COG *database. The individual *COGs *are constructed by grouping putative orthologous by bidirectional best hit in completely sequenced genomes [30]. Therefore, we think using *COGs *instead of individual proteins is less prone to classification errors. We tested *BEA *ordering algorithm by calibrating the cutting points by giving the functional category as an input. The functional category of a cluster is calculated by density (the majority of the elements in the cluster that have the same function). *BEA *accuracy was close to 99.90% of correct classification (3307 elements in the existing 18 categories). In effect, 3304 out of 3307 *COGs *were classified satisfactorily in each of the 18 existing categories. In contrast *k*-means classified 30.15% correctly, Hierarchical Clustering obtained 20.38% and *PAM *made 3.33% resulting in a notorious better accuracy for *BEA*.

In our previous studies we showed that *COG0611 *(Protein ThiL with Functional Classification H) and *COG1564 *(Protein Thi80 with Functional Classification H) are non homolog proteins with similar functions [15]. In our present study we show that they are related by *COG0352 *(Protein Thiamine monophosphate synthase with Functional Classification H). This means that *COG0611 *and *COG1564 *were close and bonded by *COG0352*. This demonstrates that *BEA *ordering algorithm worked as expected, locating functionally related proteins close together and separating unrelated proteins. However, we expected a diminishing of the accuracy of results as we removed the functional categories as an input to *BEA *ordering algorithm.

### Validations with DIP

For the second testing, we used *DIP *(Database of Interacting Proteins, Additional file 1). Particularly, 154 protein relationships were used for validations of the different methods [31]. The *DIP *database describes experimentally determined physical interactions between pairs of proteins. Therefore, in this validation we consider that two proteins are related if they belong to the same cluster. If the *DIP *relationship is contained in the same cluster then it is presumed to be a true positive, otherwise the relationship was considered a false negative. A caveat of using *DIP *is that in this database proteins that do not belong to the same functional cluster could physically interact. We also verified if the relationship was close to the neighbor clusters, maximum five, in order to calculate the efficiency of the algorithm. *BEA *accuracy in classification was approximately 62.37% in the same cluster (97 out of 154 *DIP *relationships were correctly classified). In contrast *k*-means classified 46.10% correctly, Hierarchical Clustering obtained 22.73% and *PAM *made 21.43% resulting in a notorious better accuracy for *BEA*.

The comparisons for surrounding clusters are shown in Tables 2, 3 and 4 (Additional file 4).

**Table 2:** Validation 1 for DIP in the same cluster

ALGORITHM	CORRECT	INCORRECT
BEA	62.37662	37.62338
K-MEANS	40.09091	59.90909
HIERARCHICAL	22.77922	77.22078
PAM	8.41558	91.58442

Classification for Database of Interacting Proteins.

**Table 3:** Validation 2 for DIP in the near cluster

ALGORITHM	CORRECT	INCORRECT
BEA	71.428571	28.571429
K-MEANS	46.1038961	53.8961039
HIERARCHICAL	22.7272727	77.2727273
PAM	21.4285714	78.5714286

Classification for Database of Interacting Proteins. Neighbor Cluster Maximum 1.

**Table 4:** Validation 3 for DIP in the surrounding five clusters

ALGORITHM	CORRECT	INCORRECT
BEA	86.3636364	13.6363636
K-MEANS	57.7922078	42.2077922
HIERARCHICAL	40.9090909	59.0909091
PAM	55.1948052	44.8051948

Classification for Database of Interacting Proteins. Neighbor Cluster Maximum 5.

### Validations with ECOCYC

For this test, we used the *ECOCYC *database. Specifically, 192 protein relationships were used [31] (Additional file 2). The *ECOCYC *database shows the relationships between pairs of proteins in *E. coli*. In this validation, we considered that two proteins are related if they belong to the same cluster. If the *ECOCYC *relationship was contained in the same cluster then it was presumed to be a true positive, otherwise this relationship was considered a false negative, as before. We also verified if the relationship is contained in the neighboring clusters using a radius of maximum 5 clusters, in order to analyze the efficiency of the algorithm. Under this validation, 84.37% of the entire data set was classified correctly in the same cluster (162 out of 192 *ECOCYC *relationships were clustered correctly). Compared with the other methods *k*-means classified 50.00% correctly, Hierarchical Clustering obtained 37.50% and *PAM *made 5.73% resulting again in a higher accuracy for *BEA*. The comparisons for surrounding clusters are shown in Tables 5, 6 and 7 (Additional file 5).

**Table 5:** Validation 1 for ECOCYC in the same cluster

ALGORITHM	CORRECT	INCORRECT
BEA	84.375	15.625
K-MEANS	50.00	50.00
HIERARCHICAL	37.50	62.50
PAM	5.7291667	94.2708333

Classification for Database ECOCYC.

**Table 6:** Validation 2 for ECOCYC in the near cluster

ALGORITHM	CORRECT	INCORRECT
BEA	88.5416667	11.4583333
K-MEANS	52.0833333	47.9166667
HIERARCHICAL	38.0208333	61.9791667
PAM	15.625	84.375

Classification for Database ECOCYC. Neighbor Cluster Maximum 1.

**Table 7:** Validation 3 for ECOCYC in the surrounding five clusters

ALGORITHM	CORRECT	INCORRECT
BEA	95.8333333	4.1666667
K-MEANS	65.625	34.375
HIERARCHICAL	46.3541667	53.6458333
PAM	47.9166667	52.0833333

Classification for Database ECOCYC. Neighbor Cluster Maximum 5.

As can be seen in this testing, *BEA *has a higher accuracy in classifying relationships between phylogenetic profiles. However, the capability of *BEA *was not totally exploited as we discuss in the next section.

## Discussion

**Figure 1:**
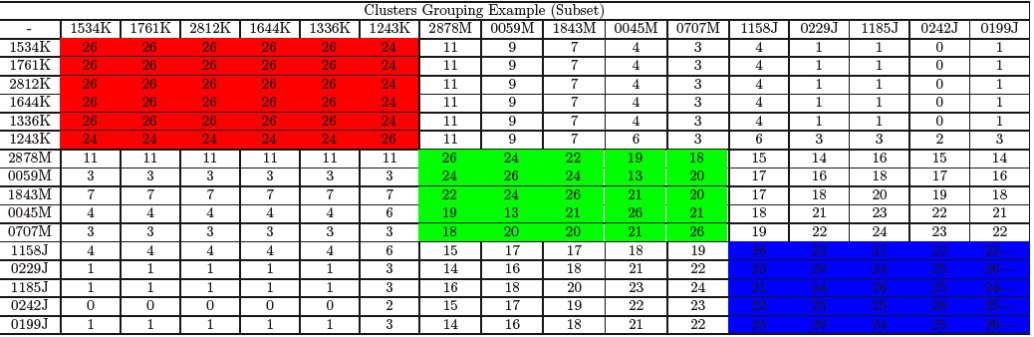
**Example of Bea Cluster**. This figure shows an example of the clusters of BEA clustering.

**Figure 2:**
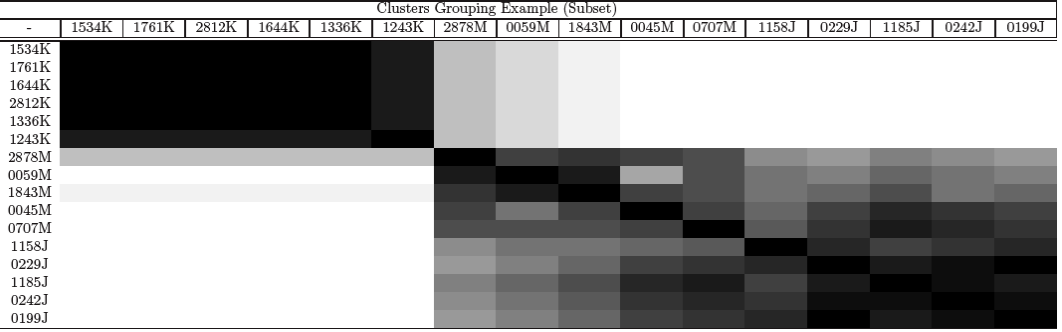
**Example of BEA clustering heatmap**. This figure shows an example of a heatmap of BEA clustering.

As shown, the validation with *COG *functional categories obtained a high classification accuracy. However, the purpose of this test was to exclusively validate *BEA *ordering algorithm in order to calibrate the cutting points. This calibration may be seen as a "guilty by association" algorithm based on *COG*'s functional categories. The next two validations were more important, as they were used to test both *BEA *ordering algorithm and to analyze *BEA *grouping algorithm (i. e. the overall performance and organizing of the algorithm. See Figure 1 and 2, Additional file 3).

We compared the three validations and analyzed the data set of the *DIP *and *ECOCYC *to understand the grouping obtained by our method. The analysis concluded that for *DIP*, the 57.80% of the relationship of these proteins are in the same functional category and 42.21% in a different category. This shows us that *BEA *is classifying reasonably well even when the proteins belong to a different functional category, although our method was used for functional category classification. For the *ECOCYC *classification we found that 84.375% belong to the same functional category and 15.625% are in a different functional category and BEA found exactly the same result.

One interesting aspect to emphasize is that there should exists several useful classification criteria depending on the abstraction level in the conceptual hierarchy of the biology of organisms to be observed. For example, at the metabolic pathway level a proper classification would include proteins with different functional categories in the same cluster. Therefore, if we perform classifications based exclusively on functional categories then the metabolic pathways would be hardly revealed by this method.

**Figure 3:**
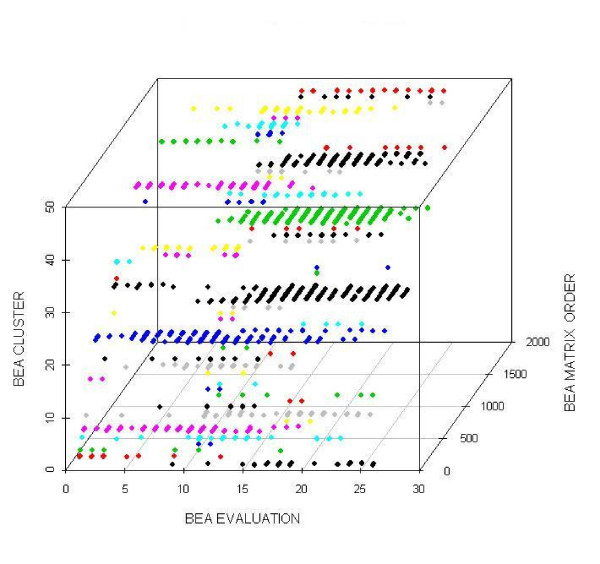
**Bea Cluster**. This figure show the distribution of the clusters for BEA.

Also we analyzed the cluster distribution and concluded that it is well balanced. This implies that the elements of the data set are well distributed among all the clusters as can be seen in Figure 3. In the case of the same functional category relationships, we found that *COG0611 *(Protein ThiL with Functional Category H) and *COG1564 *(Protein Thi80 with Functional Category H) are in the same cluster and they are related by *COG0352 *(Protein Thiamine monophosphate synthase with Functional Category H). This shows that when proteins belong to the same functional category, then they will be located close together in the *CA *matrix. Also, we found that the relationship of *COG3052 *(Protein ThiE with Functional Category H), and *COG1060 *(Protein Thi4 with Functional Category H) is very strong and this is because they participate in thiamin biosynthesis as shown in our previous work [15]. However, for those proteins that belong to different functional category even thought they are related (analogous proteins) [15] could not be accurately classified from phylogenetic profiles. For example, *COG2225 *(Protein ThiN with Functional Category C belonging to cluster 11), *COG0352 *(Protein ThiE with Functional Category H belonging to cluster 15), and *COG1992 *(Protein MTH861 with Functional Category S belonging to cluster 19). Another example is *COG02022 *(Protein ThiG with Functional Category F belonging to cluster 14), and *COG1635 *(Protein Thi4 with Functional Category R belonging to cluster 18). And also, *COG1060 *(Protein ThiH with Functional Category HR belonging to cluster 15), *COG1635 *(Protein Thi4 with Functional Category R belonging to cluster 18), and *COG0665 *(Protein ThiO with Functional Category E belonging to cluster 13). But in this case, we found that they are classified in the surrounding clusters. As can be seen the relationship between analogous proteins must be classified considering the surrounding clusters.

We have not intended our validations to be exhaustive by using all available databases. However, the consideration of additional databases such as GO, Funcat, among others, would be useful for more comprehensive validations.

## Conclusion

The focus of this study was to improve the prediction capabilities of phylogenetic profiles using *BEA*. Our results showed that *BEA *increases the accuracy of predictions of protein modules with respect to the traditional clustering methods, especially when the underlying phylogenetic patterns are relatively dissimilar. In effect, *BEA *was capable to detect relationships among proteins by relying on the presence or absence of third party proteins. This method is capable of finding relationships such as: if a protein *A *works with protein *C *and protein *B *is an analog of *A *then *A *and *B *will be related though *C*. So *A *and *B *will be functionally equivalent while *A *and *C*, and *B *and *C *will be functionally linked. This study shows that taking into account indirect relationships can be useful for detecting associations between proteins and reconstructing functional modules. If additional criteria is provided, as genomic context information, to classify different levels of relationships, higher level of accuracy could be achieved using *BEA*. Therefore, it will be useful to complement the information provided by phylogenetic profiles with additional genomic context information, such as intergenic distance and experimental data as implemented in GeConT [32].

This information can be included in the *AA *matrix to create clusters. *BEA *may also be improved by using different criteria for separating clusters to increase the capabilities of the algorithm for detecting genetic circuits for system biology [33]. On the one hand, *BEA *has many advantages, such as low processing time and memory requirements. By using the information of the attribute affinity matrix, direct and indirect relationships are considered, so this creates a balanced and more accurate classification. Based on the results presented here, we showed that *BEA *holds much promise to create better classifications for protein relationships and gene function annotation. A limitation of *BEA *from the computational perspective is the greedy nature of the algorithm, such that results are sensitive to the order of the input data. This problem could be solved by using a Genetic Algorithms to optimize the search of the solution space [34,35].

## Methods

### Data sets

We employed a collection of different data sets for conducting the experiments and validations reported here.

#### Clusters of Orthologous Groups (COG) phylogenetic patterns [30]

This data set consists of a phylogenetic classification of proteins encoded in complete genomes. *COGs *were identified by comparison of protein sequences from 43 complete genomes, representing 30 major phylogenetic lineages.

In order to extract the maximum amount of information from the rapidly accumulating genome sequences, all conserved genes need to be classified according to their homologous relationships. Comparison of proteins encoded in seven completely sequenced genomes from five major phylogenetic lineages and elucidation of consistent patterns of sequence similarities allowed the delineation of 720 clusters of orthologous groups (*COGs*).

Specifically, each *COG *consists of individual orthologous proteins or set of orthologous from at least three lineages. Orthologous typically have the same function, allowing transfer of functional information from one member to an entire *COG*. This relation automatically yields a number of functional predictions for poorly characterized genomes. The *COGs *comprise a framework for functional and evolutionary genome analysis [23,24]. In this classification, the protein group is classified by functional category, as shown in Table 8.

**Table 8:** COG FUNCTIONAL CATEGORIES

Code	COGs	Domains	Description	Pathways and functional systems
Information storage and processing				
J	217	6449	Translation, ribosomal structure and biogenesis	4
K	132	5438	Transcription	3
L	184	5337	DNA replication, recombination and repair	2
Cellular processes				
D	32	842	Cell division and chromosome partitioning	-
O	110	3165	Posttranslational modification, protein turnover, chaperones	-
M	155	4079	Cell envelope biogenesis, outer membrane	1
N	133	3110	Cell motility and secretion	2
P	160	5112	Inorganic ion transport and metabolism	1
T	97	3627	Signal transduction mechanisms	-
Metabolism				
C	224	5594	Energy production and conversion	7
G	171	5262	Carbohydrate transport and metabolism	4
E	233	8383	Amino acid transport and metabolism	10
F	85	2364	Nucleotide transport and metabolism	5
H	154	4057	Coenzyme metabolism	11
I	75	2609	Lipid metabolism	2
Q	62	2754	Secondary metabolites biosynthesis, transport and catabolism	-
Poorly characterized				
R	449	11948	General function prediction only	-
S	750	6416	Function unknown	-

Classification for COG's Database of Protein Functional Category.

#### Database of Interacting Proteins (DIP) [36]

The *DIP *database catalogs experimentally determined interactions between proteins. It combines information from a variety of sources to create a single, consistent set of protein-protein interactions. The data stored within the *DIP *database were curated, both, manually by expert curators and also automatically using computational approaches that utilize the knowledge about the protein-protein interaction networks extracted from the most reliable, core subset of the *DIP *data [37,38].

#### ECOCYC [39]

*EcoCyc *is a bioinformatics database that describes the genome and the biochemical machinery of *E. coli K-12 MG1655*. The long-term goal of the project is to describe the molecular catalog of the *E. coli *cell, as well as the functions of each of its molecular parts, to facilitate a system-level understanding of *E. coli*. *EcoCyc *is an electronic reference source for *E. coli *biologists, and for biologists who work with related microorganisms.

*EcoCyc *contains the complete genome sequence of *E. coli*, and describes the nucleotide position and function (if known) of every *E. coli *gene. A staff of five fulltime curators update the annotation of the *E. coli *genome on an ongoing basis using a literature-based curation strategy. Users can retrieve the nucleotide sequence of a gene, and the amino-acid sequence of a gene product.

*EcoCyc *describes all known metabolic pathways and signal-transduction pathways of *E. coli*. it describes each metabolic enzyme of *E. coli*, including its cofactors, activators, inhibitors, and subunit structure [40,41].

### Algorithms

The Bond Energy Algorithm (*BEA*) has been widely used for vertical fragmentation of distributed databases. This algorithm was originally proposed by McCormick and Hoffer and Severande [42]. *BEA *creates clusters using a non-trivial similarity metric (attribute affinity measure) defined on the elements of the data set. In consequence, diffcult relationships in which a third element is needed to make the relationship obvious can be discovered by this method (e. g. transitive relationships).

*BEA *is comprised by two algorithms, the first one is used for ordering the data set to locate the most related elements close together (and to separate the unrelated elements) and the second one is used for creating the groups to determine at what point has to make a cut on the ordered data set (i.e. create a cluster).

#### The BEA Ordering Algorithm

The fundamental task in designing a distributed databases vertical fragmentation algorithm is to find some means of grouping the attributes of a relation table) based on the attribute affinity values in *AA *(Attribute Affinity Matrix). It has been suggested by [43] and [44] that (*BEA*) [42] should be used for this purpose. It is considered appropriate for the following reasons [43]:

1. It is designed specifically to determine groups of similar items and opposed to, say, a linear ordering of the items (i.e., it clusters the attributes with large affinity values together, and the ones with smaller values together).

2. The final groupings are insensitive to the order in which items are presented to the algorithm.

3. The algorithm complexity is similar to other methods but can have better results [*O*(*n*2), where *n *is the number of attributes].

4. Secondary interrelationships between clustered attribute groups are identifiable.

The bond energy algorithm takes as input the attribute affinity matrix, permutes its rows and columns, and generates a clustered affinity matrix (*CA*). The permutation is done in such a way as to maximize the following global affinity measure (*AM*):

$$AM=\sum_{i=1}^{n}\sum_{j=1}^{n}aff(A_{i},A_{j})[aff(A_{i},A_{j-1})+aff(A_{i},A_{j+1})+aff(A_{i-1},A_{j})+aff(A_{i+1},A_{j})]$$

where

*aff*(*A*_0_, *A*_*j*_) = *aff*(*A*_*i*_, *A*_0_) = *aff*(*A*_*n*+1_, *A*_*j*_) = *aff*(*A*_*i*_, *A*_*n*+1_) = 0

The last set of conditions takes care of the cases where an attribute is being placed in *CA *to the left of the leftmost attribute or to the right of the rightmost attribute during column permutations, and prior to the topmost row and following the last row during row permutations. In these cases, we take 0 to be the *aff *values between the attributes being considered for placement and its left or right (top or bottom) neighbors, which do not exist in *CA*.

The maximization function considers the nearest neighbors only, thereby resulting in the grouping of large values with large ones, and small values with small ones. Also, the attribute affinity matrix (*AA*) is symmetric, because is a matrix of all element of the data set similarity values, which reduces the objective function of the formulation above to:

$$AM=\sum_{i=1}^{n}\sum_{j=1}^{n}aff(A_{i},A_{j})[aff(A_{i},A_{j-1})+aff(A_{i},A_{j+1})]$$

where *A*_*i *_is an attribute of the relation, *AA *is the Attribute Affinity Matrix, *CA *is the Clustered Affinity Matrix, and *AM *is the Affinity Measure.

The generation of the clustered affinity matrix (*CA*) is hold in three steps:

1. *Initialization*. Place and fix one of the columns of *AA *arbitrarily into *CA*. Column 1 was chosen in the algorithm.

2. *Iteration*. Pick each of the remaining *n*-*i *columns (where *i *is the number of columns already placed in *CA*) and try to place them in the remaining *i *+ 1 positions in the *CA *matrix. Choose the placement that makes the greatest contribution to the global affinity measure described above. Continue this step until no more columns remain to be placed.

3. *Row ordering*. Once the column ordering is determined, the placement of the rows should also be reordered to make their relative positions match the position of the columns.

For the second step of the algorithm to work, we need to define what is meant by the contribution of an profile to the affinity measure. This contribution can be derived as follows. Recall that the global affinity measure *AM *was previously defined as

$$AM=\sum_{i=1}^{n}\sum_{j=1}^{n}aff(A_{i},A_{j})[aff(A_{i},A_{j-1})+aff(A_{i},A_{j+1})]$$

which can be rewritten as:

$$\begin{array}{c} AM=\sum_{i=1}^{n}\sum_{j=1}^{n}[aff(A_{i},A_{j})aff(A_{i},A_{j-1})+aff(A_{i},A_{j})aff(A_{i},A_{j+1})]=\\ \sum_{i=1}^{n}[\sum_{j=1}^{n}\left[aff(A_{i},A_{j})aff(A_{i},A_{j-1}\right)+\sum_{j=1}^{n}aff(A_{i},A_{j})aff(A_{i},A_{j+1})] \end{array}$$

Let us define the bond between two attributes *A*_*x *_and *A*_*y *_as

$$bond(A_{x},A_{y})=\sum_{z=1}^{n}aff(A_{z},A_{x})aff(A_{z},A_{y})$$

This is where *BEA *find indirect relationships.

Then *AM *can be written as:

$$AM=\sum_{j=1}^{n}\left[bond(A_{j},A_{j-1})+bond(A_{j},A_{j+1})\right]$$

Now consider the following *n *attributes:

*A*_1_*A*_2_...*A*_*i*-1_*A*_*i*_*A*_*j*_*A*_*j*+1_...*A*_*n *_*AM*' = [*A*_1_*A*_2_...*A*_*i*-1_] *AM" *= [*A*_*j*+1_...*A*_*n*_]

The global affinity measure for these attributes can be written as:

$$\begin{array}{c} AM_{old}=AM^{\prime }+AM^{\prime \prime }+bond(A_{i-1},A_{i})+bond(A_{j},A_{i})bond(A_{j},A_{j+1})=\\ \sum_{l=1}^{n}\left[bond(A_{l},A_{l-1})+bond(A_{l},A_{l+1})\right] \end{array}$$

Now we consider placing a new attribute *A*_*k *_and *A*_*j *_in the clustered affinity matrix. The new global affinity measure can be similarly written as:

*AM*_*new *_= *AM' *+ *AM" *+ *bond*(*A*_*i*_, *A*_*k*_) + *bond*(*A*_*k*_, *A*_*i*_) + *bond*(*A*_*k*_, *A*_*j*_) + *bond*(*A*_*j*_, *A*_*k*_) = *AM' *+ *AM" *+ 2*bond*(*A*_*i*_, *A*_*k*_) - 2*bond*(*A*_*k*_, *A*_*j*_)

where *AM' *is the Affinity Measure before the insert position, *AM" *is the Affinity Measure after the insert position, *bond*(*A*_*i*_, *A*_*k*_) is the Bond Energy Evaluation for the insertion of the elements, and *bond*(*A*_*k*_, *A*_*j*_) is the Bond Energy Evaluation for the separation of the elements.

Thus, the next contribution to the global affinity measure of placing attribute *A*_*k *_between *A*_*i *_and *A*_*j *_is:

*cont*(*A*_*i*_, *A*_*k*_, *A*_*j*_) = *AM*_*new *_- *AM*_*old *_= *bond*(*A*_*i*_, *A*_*k*_) + *bond*(*A*_*k*_, *A*_*j*_) - *bond*(*A*_*i*_, *A*_*j*_)

The input data for our experiments were Phylogenetic Profiles, this means that the entries are strings of *1's *and *0's *that shows the presence or absence of a certain protein in some organisms, in which every column represented a gene and every row was an organism. The Hamming distance between pairs of phylogenetic profiles was used to calculate the entries of the Attribute Affinity Matrix (*AA*) that represents the relationship between proteins.

Then we run the algorithm as follows:

**input**: Phylogenetic Profiles.

**output**: *CA*: Clustered Affinity matrix and order list array.

begin

   [initialize; the AA matrix is created]

   *CA*(•, 1) ← AA(•, 1)

   CA(•, 2) ← AA(•, 2)

   *index *← 3

   **while ***index *≤ *n ***do **[choose the "best" location for profile *AA*_*index*_]

   **begin**

      **for ***i ***from **1 **to ***index *- 1 **by **1 **do**

         calculate *cont*(*AA*_*i*-1_, *AA*_*index*_, *AA*_*i*_)

      **end-for**

      calculate *cont*(*AA*_*index*-1_, *AA*_*index*_, *AA*_*index*+1 _[boundary condition]

      *loc *← placement given by maximum *cont *value

      **for ***j ***from ***index ***to ***loc ***by **-1 **do **[shuffle the two matrices]

         calculate *CA*(•, j) ← *CA*(•, *j *- 1)

      **end-for**

      *CA*(•, *loc*) ← *AA*(•, *index*)

      *index *← *index *+ 1

   **end-while**

   order the rows according to the relative ordering of columns

**end**.

Note: • means for each element in the data set.

#### The BEA Grouping Algorithm

Once *BEA *ordering algorithm was executed on the input data, the *CA *matrix must be grouped, for this propose we used a "guilty by association" method based on *COG*'s functional categories. Particularly, the unknown function of a protein was transferred from the neighbor proteins with characterized function in the *CA*[45,46].

Especifically,

if *i *+ 1 and *i *- 1 category is known and *category*(*i *- 1) is equal to *category*(*i *+ 1) then *category*(*i*) = *category*(*i *- 1)

where *i *is the position in the ordered matrix.

The cutting point is calculated when a change in the classification occurs.

We repeat the cut process until reaching the total number of elements in the *CA *matrix.

### Validations

Our validations were made using the above mentioned data sets: the *COG's *functional categories for testing *BEA *ordering algorithm; and the *DIP *and *ECOCYC *databases were used as additional data sets for testing accuracy of *BEA *grouping algorithm.

## Authors' contributions

EM suggested the biological model. RLAW and EEV suggested the use of BEA. RLAW implemented the algorithm and performed the computational experiments. RLAW, EEV, and EM wrote the manuscript. All authors contributed equally in this research. All authors read and approved the final manuscript.

## Supplementary Material

Additional file 1 Table of DIP Relationships used for validation. Click here for file

Additional file 4 DIP validation table. Click here for file

Additional file 2 Table of ECOCYC Relationships used for validation. Click here for file

Additional file 5 ECOCYC validation table. Click here for file

Additional file 3 Result of BEA cluster. Click here for file
