# Supplementary material for: Inferring modules of functionally interacting proteins using the Bond Energy Algorithm
Source: BMC Bioinformatics. 2008 Jun 17;9:285. doi: 10.1186/1471-2105-9-285 (PMC2474619; doi:10.1186/1471-2105-9-285)
Supplement: Additional file 1 — Table of DIP Relationships used for validation. [file 1471-2105-9-285-S1.pdf]

ANNEX 1: RELATIONSHIPS OF DATABASE OF INTERACTING PROTEINS

Database of Interacting Proteins (DIP)		
1	COG0029	COG0379
2	COG0045	COG0074
3	COG0050	COG0264
4	COG0055	COG0056
5	COG0055	COG0224
6	COG0055	COG0356
7	COG0055	COG0711
8	COG0055	COG0712
9	COG0056	COG0224
10	COG0056	COG0711
11	COG0056	COG0712
12	COG0085	COG0086
13	COG0085	COG0195
14	COG0085	COG0202
15	COG0086	COG0195
16	COG0086	COG0202
17	COG0086	COG0568
18	COG0086	COG1595
19	COG0086	COG3711
20	COG0133	COG0159
21	COG0147	COG0512
22	COG0148	COG1185
23	COG0148	COG1530
24	COG0175	COG2895
25	COG0187	COG3449
26	COG0188	COG3449
27	COG0195	COG0202
28	COG0201	COG0653
29	COG0201	COG0690
30	COG0201	COG1314
31	COG0201	COG1589
32	COG0202	COG0664
33	COG0206	COG0849
34	COG0206	COG3115
35	COG0208	COG0209
36	COG0210	COG0249
37	COG0210	COG0323
38	COG0224	COG0355
39	COG0224	COG0712
40	COG0249	COG0323
41	COG0249	COG3066
42	COG0250	COG1158
43	COG0282	COG1080
44	COG0303	COG0521
45	COG0303	COG0746
46	COG0303	COG1763
47	COG0305	COG0358
48	COG0305	COG0593

49	COG0305	COG1484
50	COG0316	COG0443
51	COG0316	COG0633
52	COG0323	COG3066
53	COG0323	COG3727
54	COG0347	COG3852
55	COG0353	COG1381
56	COG0355	COG0712
57	COG0356	COG0711
58	COG0389	COG1974
59	COG0419	COG0420
60	COG0443	COG0484
61	COG0443	COG0568
62	COG0443	COG0576
63	COG0443	COG0633
64	COG0443	COG0822
65	COG0443	COG1076
66	COG0443	COG1185
67	COG0443	COG1530
68	COG0458	COG0505
69	COG0468	COG1974
70	COG0468	COG2137
71	COG0479	COG1053
72	COG0479	COG2009
73	COG0479	COG2142
74	COG0479	COG3029
75	COG0479	COG3080
76	COG0484	COG0568
77	COG0492	COG0526
78	COG0513	COG1185
79	COG0513	COG1530
80	COG0521	COG1763
81	COG0540	COG1781
82	COG0542	COG0740
83	COG0542	COG2127
84	COG0554	COG2190
85	COG0583	COG2716
86	COG0629	COG1381
87	COG0629	COG2925
88	COG0632	COG2255
89	COG0633	COG1104
90	COG0642	COG0745
91	COG0642	COG0784
92	COG0642	COG2197
93	COG0643	COG0784
94	COG0643	COG3143
95	COG0653	COG1314
96	COG0653	COG1589
97	COG0653	COG1952
98	COG0657	COG1486
99	COG0657	COG2909
100	COG0664	COG1609

101	COG0706	COG1589
102	COG0711	COG0712
103	COG0740	COG1219
104	COG0746	COG1763
105	COG0784	COG1868
106	COG0784	COG2197
107	COG0784	COG2198
108	COG0784	COG3143
109	COG0805	COG1826
110	COG0810	COG0811
111	COG0810	COG0848
112	COG0810	COG1629
113	COG0810	COG2885
114	COG0811	COG0848
115	COG0811	COG3064
116	COG0822	COG1076
117	COG0822	COG1104
118	COG0823	COG2885
119	COG0823	COG3064
120	COG0823	COG3203
121	COG0834	COG2197
122	COG0840	COG1352
123	COG0841	COG0845
124	COG0848	COG3064
125	COG0850	COG2894
126	COG1053	COG3029
127	COG1138	COG2332
128	COG1138	COG3088
129	COG1159	COG1694
130	COG1168	COG2909
131	COG1175	COG3839
132	COG1185	COG1530
133	COG1195	COG1381
134	COG1221	COG1842
135	COG1263	COG3711
136	COG1264	COG3711
137	COG1282	COG3288
138	COG1536	COG1766
139	COG1536	COG1868
140	COG1536	COG2916
141	COG1595	COG3026
142	COG1595	COG3073
143	COG1595	COG3712
144	COG1729	COG3064
145	COG1868	COG1886
146	COG2009	COG2142
147	COG2182	COG3833
148	COG2190	COG3711
149	COG2197	COG2198
150	COG2731	COG3250
151	COG3026	COG3073
152	COG3029	COG3080

153	COG3064	COG3203
154	COG3833	COG3839
