# Supplementary material for: Inferring modules of functionally interacting proteins using the Bond Energy Algorithm
Source: BMC Bioinformatics. 2008 Jun 17;9:285. doi: 10.1186/1471-2105-9-285 (PMC2474619; doi:10.1186/1471-2105-9-285)
Supplement: Additional file 4 — DIP validation table. [file 1471-2105-9-285-S4.pdf]

Database of Interacting Proteins (DIP)

		YES	NO	NEXT 1	NO	NEXT 2	NO	NEXT 5	NO
COG0029	COG0379	1	0	1	0	1	0	1	0
COG0045	COG0074	1	0	1	0	1	0	1	0
COG0050	COG0264	1	0	1	0	1	0	1	0
COG0055	COG0056	1	0	1	0	1	0	1	0
COG0055	COG0224	1	0	1	0	1	0	1	0
COG0055	COG0356	1	0	1	0	1	0	1	0
COG0055	COG0711	1	0	1	0	1	0	1	0
COG0055	COG0712	1	0	1	0	1	0	1	0
COG0056	COG0224	1	0	1	0	1	0	1	0
COG0056	COG0711	1	0	1	0	1	0	1	0
COG0056	COG0712	1	0	1	0	1	0	1	0
COG0085	COG0086	1	0	1	0	1	0	1	0
COG0085	COG0195	1	0	1	0	1	0	1	0
COG0085	COG0202	1	0	1	0	1	0	1	0
COG0086	COG0195	1	0	1	0	1	0	1	0
COG0086	COG0202	1	0	1	0	1	0	1	0
COG0086	COG0568	1	0	1	0	1	0	1	0
COG0086	COG1595	1	0	1	0	1	0	1	0
COG0086	COG3711	1	0	1	0	1	0	1	0
COG0133	COG0159	1	0	1	0	1	0	1	0
COG0147	COG0512	1	0	1	0	1	0	1	0
COG0148	COG1185	0	1	0	1	0	1	0	1
COG0148	COG1530	0	1	0	1	0	1	0	1
COG0175	COG2895	0	1	0	1	0	1	1	0
COG0187	COG3449	1	0	1	0	1	0	1	0
COG0188	COG3449	1	0	1	0	1	0	1	0
COG0195	COG0202	1	0	1	0	1	0	1	0
COG0201	COG0653	1	0	1	0	1	0	1	0
COG0201	COG0690	1	0	1	0	1	0	1	0
COG0201	COG1314	1	0	1	0	1	0	1	0
COG0201	COG1589	0	1	1	0	1	0	1	0
COG0202	COG0664	0	1	0	1	0	1	0	1
COG0206	COG0849	1	0	1	0	1	0	1	0
COG0206	COG3115	1	0	1	0	1	0	1	0
COG0208	COG0209	1	0	1	0	1	0	1	0
COG0210	COG0249	1	0	1	0	1	0	1	0
COG0210	COG0323	1	0	1	0	1	0	1	0
COG0224	COG0355	1	0	1	0	1	0	1	0
COG0224	COG0712	1	0	1	0	1	0	1	0
COG0249	COG0323	1	0	1	0	1	0	1	0
COG0249	COG3066	1	0	1	0	1	0	1	0
COG0250	COG1158	1	0	1	0	1	0	1	0
COG0282	COG1080	0	1	1	0	1	0	1	0
COG0303	COG0521	1	0	1	0	1	0	1	0
COG0303	COG0746	1	0	1	0	1	0	1	0
COG0303	COG1763	1	0	1	0	1	0	1	0
COG0305	COG0358	1	0	1	0	1	0	1	0
COG0305	COG0593	1	0	1	0	1	0	1	0
COG0305	COG1484	1	0	1	0	1	0	1	0
COG0316	COG0443	0	1	0	1	0	1	0	1

COG0316	COG0633	0	1	0	1	0	1	0	1
COG0323	COG3066	1	0	1	0	1	0	1	0
COG0323	COG3727	1	0	1	0	1	0	1	0
COG0347	COG3852	0	1	0	1	0	1	1	0
COG0353	COG1381	1	0	1	0	1	0	1	0
COG0355	COG0712	1	0	1	0	1	0	1	0
COG0356	COG0711	1	0	1	0	1	0	1	0
COG0389	COG1974	0	1	1	0	1	0	1	0
COG0419	COG0420	1	0	1	0	1	0	1	0
COG0443	COG0484	1	0	1	0	1	0	1	0
COG0443	COG0568	0	1	0	1	0	1	1	0
COG0443	COG0576	1	0	1	0	1	0	1	0
COG0443	COG0633	0	1	0	1	0	1	1	0
COG0443	COG0822	0	1	0	1	0	1	1	0
COG0443	COG1076	1	0	1	0	1	0	1	0
COG0443	COG1185	0	1	0	1	0	1	1	0
COG0443	COG1530	0	1	0	1	0	1	1	0
COG0458	COG0505	1	0	1	0	1	0	1	0
COG0468	COG1974	0	1	1	0	1	0	1	0
COG0468	COG2137	0	1	0	1	0	1	0	1
COG0479	COG1053	1	0	1	0	1	0	1	0
COG0479	COG2009	1	0	1	0	1	0	1	0
COG0479	COG2142	1	0	1	0	1	0	1	0
COG0479	COG3029	1	0	1	0	1	0	1	0
COG0479	COG3080	1	0	1	0	1	0	1	0
COG0484	COG0568	0	1	0	1	0	1	1	0
COG0492	COG0526	1	0	1	0	1	0	1	0
COG0513	COG1185	0	1	0	1	1	0	1	0
COG0513	COG1530	0	1	0	1	1	0	1	0
COG0521	COG1763	1	0	1	0	1	0	1	0
COG0540	COG1781	1	0	1	0	1	0	1	0
COG0542	COG0740	0	1	0	1	1	0	1	0
COG0542	COG2127	0	1	0	1	0	1	0	1
COG0554	COG2190	0	1	1	0	1	0	1	0
COG0583	COG2716	0	1	0	1	0	1	0	1
COG0629	COG1381	1	0	1	0	1	0	1	0
COG0629	COG2925	1	0	1	0	1	0	1	0
COG0632	COG2255	1	0	1	0	1	0	1	0
COG0633	COG1104	0	1	0	1	1	0	1	0
COG0642	COG0745	1	0	1	0	1	0	1	0
COG0642	COG0784	1	0	1	0	1	0	1	0
COG0642	COG2197	1	0	1	0	1	0	1	0
COG0643	COG0784	0	1	0	1	1	0	1	0
COG0643	COG3143	1	0	1	0	1	0	1	0
COG0653	COG1314	1	0	1	0	1	0	1	0
COG0653	COG1589	0	1	1	0	1	0	1	0
COG0653	COG1952	1	0	1	0	1	0	1	0
COG0657	COG1486	0	1	0	1	0	1	1	0
COG0657	COG2909	0	1	0	1	0	1	0	1
COG0664	COG1609	0	1	0	1	0	1	0	1
COG0706	COG1589	0	1	1	0	1	0	1	0
COG0711	COG0712	1	0	1	0	1	0	1	0

COG0740	COG1219	0	1	0	1	1	0	1	0
COG0746	COG1763	1	0	1	0	1	0	1	0
COG0784	COG1868	0	1	0	1	1	0	1	0
COG0784	COG2197	1	0	1	0	1	0	1	0
COG0784	COG2198	1	0	1	0	1	0	1	0
COG0784	COG3143	0	1	0	1	1	0	1	0
COG0805	COG1826	1	0	1	0	1	0	1	0
COG0810	COG0811	0	1	1	0	1	0	1	0
COG0810	COG0848	0	1	1	0	1	0	1	0
COG0810	COG1629	0	1	0	1	1	0	1	0
COG0810	COG2885	1	0	1	0	1	0	1	0
COG0811	COG0848	1	0	1	0	1	0	1	0
COG0811	COG3064	0	1	1	0	1	0	1	0
COG0822	COG1076	0	1	0	1	0	1	1	0
COG0822	COG1104	0	1	0	1	1	0	1	0
COG0823	COG2885	0	1	1	0	1	0	1	0
COG0823	COG3064	0	1	1	0	1	0	1	0
COG0823	COG3203	0	1	1	0	1	0	1	0
COG0834	COG2197	0	1	0	1	0	1	1	0
COG0840	COG1352	1	0	1	0	1	0	1	0
COG0841	COG0845	1	0	1	0	1	0	1	0
COG0848	COG3064	0	1	1	0	1	0	1	0
COG0850	COG2894	1	0	1	0	1	0	1	0
COG1053	COG3029	1	0	1	0	1	0	1	0
COG1138	COG2332	1	0	1	0	1	0	1	0
COG1138	COG3088	1	0	1	0	1	0	1	0
COG1159	COG1694	1	0	1	0	1	0	1	0
COG1168	COG2909	0	1	0	1	0	1	0	1
COG1175	COG3839	0	1	0	1	0	1	1	0
COG1185	COG1530	1	0	1	0	1	0	1	0
COG1195	COG1381	1	0	1	0	1	0	1	0
COG1221	COG1842	0	1	0	1	0	1	0	1
COG1263	COG3711	0	1	0	1	0	1	0	1
COG1264	COG3711	0	1	0	1	0	1	0	1
COG1282	COG3288	1	0	1	0	1	0	1	0
COG1536	COG1766	1	0	1	0	1	0	1	0
COG1536	COG1868	1	0	1	0	1	0	1	0
COG1536	COG2916	0	1	0	1	0	1	0	1
COG1595	COG3026	0	1	0	1	0	1	0	1
COG1595	COG3073	0	1	0	1	0	1	0	1
COG1595	COG3712	0	1	0	1	0	1	0	1
COG1729	COG3064	0	1	0	1	0	1	0	1
COG1868	COG1886	1	0	1	0	1	0	1	0
COG2009	COG2142	1	0	1	0	1	0	1	0
COG2182	COG3833	1	0	1	0	1	0	1	0
COG2190	COG3711	0	1	0	1	0	1	0	1
COG2197	COG2198	1	0	1	0	1	0	1	0
COG2731	COG3250	0	1	0	1	0	1	0	1
COG3026	COG3073	1	0	1	0	1	0	1	0
COG3029	COG3080	1	0	1	0	1	0	1	0
COG3064	COG3203	1	0	1	0	1	0	1	0
COG3833	COG3839	0	1	0	1	0	1	1	0

96	58	110	44	120	34	133	21
0.62	0.4	0.7143	0.3	0.7792	0.2	0.8636	0.1
