# Supplementary material for: Inferring modules of functionally interacting proteins using the Bond Energy Algorithm
Source: BMC Bioinformatics. 2008 Jun 17;9:285. doi: 10.1186/1471-2105-9-285 (PMC2474619; doi:10.1186/1471-2105-9-285)
Supplement: Additional file 2 — Table of ECOCYC Relationships used for validation. [file 1471-2105-9-285-S2.pdf]

ANNEX 2: ECOCYC RELATIONSHIPS

ECOCYC		
1	COG0001	COG0113
2	COG0001	COG0373
3	COG0002	COG0548
4	COG0007	COG1587
5	COG0010	COG1166
6	COG0015	COG0104
7	COG0015	COG0138
8	COG0015	COG0152
9	COG0015	COG0563
10	COG0019	COG0253
11	COG0020	COG0142
12	COG0026	COG0041
13	COG0026	COG0150
14	COG0027	COG0046
15	COG0027	COG0047
16	COG0027	COG0151
17	COG0034	COG0151
18	COG0040	COG0139
19	COG0040	COG0140
20	COG0041	COG0152
21	COG0043	COG0382
22	COG0043	COG0661
23	COG0046	COG0299
24	COG0047	COG0150
25	COG0047	COG0299
26	COG0054	COG0108
27	COG0054	COG0307
28	COG0058	COG1640
29	COG0059	COG0440
30	COG0065	COG0473
31	COG0066	COG0473
32	COG0078	COG0137
33	COG0079	COG0131
34	COG0079	COG0241
35	COG0082	COG0128
36	COG0083	COG0498
37	COG0104	COG0138
38	COG0106	COG0107
39	COG0106	COG0118
40	COG0106	COG0139
41	COG0106	COG0140
42	COG0107	COG0131
43	COG0107	COG0241
44	COG0109	COG0276
45	COG0111	COG1932
46	COG0113	COG0181
47	COG0117	COG0807
48	COG0118	COG0131

49	COG0118	COG0241
50	COG0126	COG0588
51	COG0126	COG0696
52	COG0128	COG0703
53	COG0131	COG0141
54	COG0132	COG0161
55	COG0132	COG0502
56	COG0133	COG0134
57	COG0133	COG0135
58	COG0134	COG0159
59	COG0134	COG0547
60	COG0135	COG0159
61	COG0135	COG0547
62	COG0136	COG0527
63	COG0137	COG0165
64	COG0138	COG0516
65	COG0141	COG0241
66	COG0142	COG1443
67	COG0151	COG0299
68	COG0155	COG0175
69	COG0157	COG0379
70	COG0163	COG0382
71	COG0163	COG0661
72	COG0167	COG0418
73	COG0167	COG0461
74	COG0169	COG0703
75	COG0169	COG0710
76	COG0171	COG1057
77	COG0175	COG0369
78	COG0181	COG1587
79	COG0190	COG0285
80	COG0194	COG0518
81	COG0194	COG0519
82	COG0196	COG0307
83	COG0204	COG0575
84	COG0204	COG2937
85	COG0237	COG0669
86	COG0245	COG0821
87	COG0245	COG1947
88	COG0248	COG0317
89	COG0262	COG0285
90	COG0269	COG3623
91	COG0281	COG0574
92	COG0283	COG0572
93	COG0284	COG0461
94	COG0284	COG0528
95	COG0288	COG1513
96	COG0289	COG2171
97	COG0294	COG0801
98	COG0296	COG0297
99	COG0297	COG0448
100	COG0320	COG0321

101	COG0331	COG0332
102	COG0331	COG0439
103	COG0331	COG0511
104	COG0331	COG0777
105	COG0331	COG0825
106	COG0337	COG0710
107	COG0337	COG0722
108	COG0340	COG0439
109	COG0346	COG0491
110	COG0346	COG1803
111	COG0351	COG0352
112	COG0351	COG0422
113	COG0352	COG0611
114	COG0352	COG1060
115	COG0352	COG2022
116	COG0352	COG2145
117	COG0363	COG1820
118	COG0368	COG2087
119	COG0372	COG1049
120	COG0376	COG0605
121	COG0380	COG1877
122	COG0381	COG0677
123	COG0382	COG3161
124	COG0399	COG0454
125	COG0407	COG0408
126	COG0407	COG0635
127	COG0407	COG1893
128	COG0408	COG0853
129	COG0413	COG1893
130	COG0414	COG0853
131	COG0414	COG1893
132	COG0418	COG0540
133	COG0418	COG1781
134	COG0421	COG1586
135	COG0431	COG2141
136	COG0439	COG1654
137	COG0447	COG1575
138	COG0451	COG1089
139	COG0452	COG0669
140	COG0452	COG1072
141	COG0503	COG0813
142	COG0516	COG0518
143	COG0516	COG0519
144	COG0529	COG2895
145	COG0558	COG0575
146	COG0558	COG0671
147	COG0558	COG1267
148	COG0575	COG1502
149	COG0605	COG0753
150	COG0661	COG2227
151	COG0671	COG1502
152	COG0677	COG1922

153	COG0688	COG1502
154	COG0717	COG0756
155	COG0743	COG1211
156	COG0761	COG0821
157	COG0763	COG1044
158	COG0763	COG2908
159	COG0766	COG0812
160	COG0769	COG0770
161	COG0769	COG0771
162	COG0770	COG1181
163	COG0771	COG0773
164	COG0771	COG0796
165	COG0773	COG0812
166	COG0774	COG1043
167	COG0774	COG1044
168	COG0794	COG2877
169	COG0801	COG1539
170	COG0807	COG1985
171	COG0837	COG1626
172	COG0854	COG1995
173	COG1018	COG1541
174	COG1044	COG2908
175	COG1069	COG2160
176	COG1088	COG1209
177	COG1165	COG1441
178	COG1211	COG1947
179	COG1212	COG1519
180	COG1267	COG1502
181	COG1335	COG1488
182	COG1352	COG2201
183	COG1519	COG1663
184	COG1541	COG2151
185	COG1541	COG3396
186	COG1587	COG1648
187	COG1767	COG3697
188	COG1778	COG2877
189	COG1929	COG2084
190	COG1940	COG3010
191	COG2087	COG2109
192	COG3138	COG3724
