# Supplementary material for: Inferring modules of functionally interacting proteins using the Bond Energy Algorithm
Source: BMC Bioinformatics. 2008 Jun 17;9:285. doi: 10.1186/1471-2105-9-285 (PMC2474619; doi:10.1186/1471-2105-9-285)
Supplement: Additional file 5 — ECOCYC validation table. [file 1471-2105-9-285-S5.pdf]

| ECOCYC | | YES | NO | NEXT 1 | NO | NEXT 2 | NO | NEXT 5 | NO |
|---|---|---|---|---|---|---|---|---|---|
| COG0001 | COG0113 | 1 | 0 | 1 | 0 | 1 | 0 | 1 | 0 |
| COG0001 | COG0373 | 1 | 0 | 1 | 0 | 1 | 0 | 1 | 0 |
| COG0002 | COG0548 | 1 | 0 | 1 | 0 | 1 | 0 | 1 | 0 |
| COG0007 | COG1587 | 1 | 0 | 1 | 0 | 1 | 0 | 1 | 0 |
| COG0010 | COG1166 | 1 | 0 | 1 | 0 | 1 | 0 | 1 | 0 |
| COG0015 | COG0104 | 1 | 0 | 1 | 0 | 1 | 0 | 1 | 0 |
| COG0015 | COG0138 | 1 | 0 | 1 | 0 | 1 | 0 | 1 | 0 |
| COG0015 | COG0152 | 1 | 0 | 1 | 0 | 1 | 0 | 1 | 0 |
| COG0015 | COG0563 | 1 | 0 | 1 | 0 | 1 | 0 | 1 | 0 |
| COG0019 | COG0253 | 1 | 0 | 1 | 0 | 1 | 0 | 1 | 0 |
| COG0020 | COG0142 | 0 | 1 | 1 | 0 | 1 | 0 | 1 | 0 |
| COG0026 | COG0041 | 1 | 0 | 1 | 0 | 1 | 0 | 1 | 0 |
| COG0026 | COG0150 | 1 | 0 | 1 | 0 | 1 | 0 | 1 | 0 |
| COG0027 | COG0046 | 1 | 0 | 1 | 0 | 1 | 0 | 1 | 0 |
| COG0027 | COG0047 | 1 | 0 | 1 | 0 | 1 | 0 | 1 | 0 |
| COG0027 | COG0151 | 1 | 0 | 1 | 0 | 1 | 0 | 1 | 0 |
| COG0034 | COG0151 | 1 | 0 | 1 | 0 | 1 | 0 | 1 | 0 |
| COG0040 | COG0139 | 1 | 0 | 1 | 0 | 1 | 0 | 1 | 0 |
| COG0040 | COG0140 | 1 | 0 | 1 | 0 | 1 | 0 | 1 | 0 |
| COG0041 | COG0152 | 1 | 0 | 1 | 0 | 1 | 0 | 1 | 0 |
| COG0043 | COG0382 | 1 | 0 | 1 | 0 | 1 | 0 | 1 | 0 |
| COG0043 | COG0661 | 0 | 1 | 0 | 1 | 0 | 1 | 1 | 0 |
| COG0046 | COG0299 | 1 | 0 | 1 | 0 | 1 | 0 | 1 | 0 |
| COG0047 | COG0150 | 1 | 0 | 1 | 0 | 1 | 0 | 1 | 0 |
| COG0047 | COG0299 | 1 | 0 | 1 | 0 | 1 | 0 | 1 | 0 |
| COG0054 | COG0108 | 1 | 0 | 1 | 0 | 1 | 0 | 1 | 0 |
| COG0054 | COG0307 | 1 | 0 | 1 | 0 | 1 | 0 | 1 | 0 |
| COG0058 | COG1640 | 1 | 0 | 1 | 0 | 1 | 0 | 1 | 0 |
| COG0059 | COG0440 | 1 | 0 | 1 | 0 | 1 | 0 | 1 | 0 |
| COG0065 | COG0473 | 1 | 0 | 1 | 0 | 1 | 0 | 1 | 0 |
| COG0066 | COG0473 | 1 | 0 | 1 | 0 | 1 | 0 | 1 | 0 |
| COG0078 | COG0137 | 1 | 0 | 1 | 0 | 1 | 0 | 1 | 0 |
| COG0079 | COG0131 | 1 | 0 | 1 | 0 | 1 | 0 | 1 | 0 |
| COG0079 | COG0241 | 1 | 0 | 1 | 0 | 1 | 0 | 1 | 0 |
| COG0082 | COG0128 | 1 | 0 | 1 | 0 | 1 | 0 | 1 | 0 |
| COG0083 | COG0498 | 1 | 0 | 1 | 0 | 1 | 0 | 1 | 0 |
| COG0104 | COG0138 | 1 | 0 | 1 | 0 | 1 | 0 | 1 | 0 |
| COG0106 | COG0107 | 1 | 0 | 1 | 0 | 1 | 0 | 1 | 0 |
| COG0106 | COG0118 | 1 | 0 | 1 | 0 | 1 | 0 | 1 | 0 |
| COG0106 | COG0139 | 1 | 0 | 1 | 0 | 1 | 0 | 1 | 0 |
| COG0106 | COG0140 | 1 | 0 | 1 | 0 | 1 | 0 | 1 | 0 |
| COG0107 | COG0131 | 1 | 0 | 1 | 0 | 1 | 0 | 1 | 0 |
| COG0107 | COG0241 | 1 | 0 | 1 | 0 | 1 | 0 | 1 | 0 |
| COG0109 | COG0276 | 1 | 0 | 1 | 0 | 1 | 0 | 1 | 0 |
| COG0111 | COG1932 | 0 | 1 | 0 | 1 | 1 | 0 | 1 | 0 |
| COG0113 | COG0181 | 1 | 0 | 1 | 0 | 1 | 0 | 1 | 0 |
| COG0117 | COG0807 | 1 | 0 | 1 | 0 | 1 | 0 | 1 | 0 |
| COG0118 | COG0131 | 1 | 0 | 1 | 0 | 1 | 0 | 1 | 0 |
| COG0118 | COG0241 | 1 | 0 | 1 | 0 | 1 | 0 | 1 | 0 |
| COG0126 | COG0588 | 1 | 0 | 1 | 0 | 1 | 0 | 1 | 0 |
| COG0126 | COG0696 | 1 | 0 | 1 | 0 | 1 | 0 | 1 | 0 |
| COG0128 | COG0703 | 1 | 0 | 1 | 0 | 1 | 0 | 1 | 0 |
| COG0131 | COG0141 | 1 | 0 | 1 | 0 | 1 | 0 | 1 | 0 |
| COG0132 | COG0161 | 1 | 0 | 1 | 0 | 1 | 0 | 1 | 0 |
| COG0132 | COG0502 | 1 | 0 | 1 | 0 | 1 | 0 | 1 | 0 |

| | | | | | | | | | |
|---|---|---|---|---|---|---|---|---|---|
| COG0133 | COG0134 | 1 | 0 | 1 | 0 | 1 | 0 | 1 | 0 |
| COG0133 | COG0135 | 1 | 0 | 1 | 0 | 1 | 0 | 1 | 0 |
| COG0134 | COG0159 | 1 | 0 | 1 | 0 | 1 | 0 | 1 | 0 |
| COG0134 | COG0547 | 1 | 0 | 1 | 0 | 1 | 0 | 1 | 0 |
| COG0135 | COG0159 | 1 | 0 | 1 | 0 | 1 | 0 | 1 | 0 |
| COG0135 | COG0547 | 1 | 0 | 1 | 0 | 1 | 0 | 1 | 0 |
| COG0136 | COG0527 | 1 | 0 | 1 | 0 | 1 | 0 | 1 | 0 |
| COG0137 | COG0165 | 1 | 0 | 1 | 0 | 1 | 0 | 1 | 0 |
| COG0138 | COG0516 | 1 | 0 | 1 | 0 | 1 | 0 | 1 | 0 |
| COG0141 | COG0241 | 1 | 0 | 1 | 0 | 1 | 0 | 1 | 0 |
| COG0142 | COG1443 | 0 | 1 | 1 | 0 | 1 | 0 | 1 | 0 |
| COG0151 | COG0299 | 1 | 0 | 1 | 0 | 1 | 0 | 1 | 0 |
| COG0155 | COG0175 | 0 | 1 | 0 | 1 | 0 | 1 | 1 | 0 |
| COG0157 | COG0379 | 1 | 0 | 1 | 0 | 1 | 0 | 1 | 0 |
| COG0163 | COG0382 | 1 | 0 | 1 | 0 | 1 | 0 | 1 | 0 |
| COG0163 | COG0661 | 0 | 1 | 0 | 1 | 0 | 1 | 1 | 0 |
| COG0167 | COG0418 | 1 | 0 | 1 | 0 | 1 | 0 | 1 | 0 |
| COG0167 | COG0461 | 1 | 0 | 1 | 0 | 1 | 0 | 1 | 0 |
| COG0169 | COG0703 | 1 | 0 | 1 | 0 | 1 | 0 | 1 | 0 |
| COG0169 | COG0710 | 1 | 0 | 1 | 0 | 1 | 0 | 1 | 0 |
| COG0171 | COG1057 | 1 | 0 | 1 | 0 | 1 | 0 | 1 | 0 |
| COG0175 | COG0369 | 0 | 1 | 0 | 1 | 0 | 1 | 1 | 0 |
| COG0181 | COG1587 | 1 | 0 | 1 | 0 | 1 | 0 | 1 | 0 |
| COG0190 | COG0285 | 1 | 0 | 1 | 0 | 1 | 0 | 1 | 0 |
| COG0194 | COG0518 | 1 | 0 | 1 | 0 | 1 | 0 | 1 | 0 |
| COG0194 | COG0519 | 1 | 0 | 1 | 0 | 1 | 0 | 1 | 0 |
| COG0196 | COG0307 | 1 | 0 | 1 | 0 | 1 | 0 | 1 | 0 |
| COG0204 | COG0575 | 1 | 0 | 1 | 0 | 1 | 0 | 1 | 0 |
| COG0204 | COG2937 | 1 | 0 | 1 | 0 | 1 | 0 | 1 | 0 |
| COG0237 | COG0669 | 1 | 0 | 1 | 0 | 1 | 0 | 1 | 0 |
| COG0245 | COG0821 | 0 | 1 | 0 | 1 | 0 | 1 | 0 | 1 |
| COG0245 | COG1947 | 1 | 0 | 1 | 0 | 1 | 0 | 1 | 0 |
| COG0248 | COG0317 | 0 | 1 | 0 | 1 | 0 | 1 | 1 | 0 |
| COG0262 | COG0285 | 1 | 0 | 1 | 0 | 1 | 0 | 1 | 0 |
| COG0269 | COG3623 | 1 | 0 | 1 | 0 | 1 | 0 | 1 | 0 |
| COG0281 | COG0574 | 0 | 1 | 1 | 0 | 1 | 0 | 1 | 0 |
| COG0283 | COG0572 | 1 | 0 | 1 | 0 | 1 | 0 | 1 | 0 |
| COG0284 | COG0461 | 1 | 0 | 1 | 0 | 1 | 0 | 1 | 0 |
| COG0284 | COG0528 | 1 | 0 | 1 | 0 | 1 | 0 | 1 | 0 |
| COG0288 | COG1513 | 1 | 0 | 1 | 0 | 1 | 0 | 1 | 0 |
| COG0289 | COG2171 | 1 | 0 | 1 | 0 | 1 | 0 | 1 | 0 |
| COG0294 | COG0801 | 1 | 0 | 1 | 0 | 1 | 0 | 1 | 0 |
| COG0296 | COG0297 | 1 | 0 | 1 | 0 | 1 | 0 | 1 | 0 |
| COG0297 | COG0448 | 1 | 0 | 1 | 0 | 1 | 0 | 1 | 0 |
| COG0320 | COG0321 | 1 | 0 | 1 | 0 | 1 | 0 | 1 | 0 |
| COG0331 | COG0332 | 1 | 0 | 1 | 0 | 1 | 0 | 1 | 0 |
| COG0331 | COG0439 | 1 | 0 | 1 | 0 | 1 | 0 | 1 | 0 |
| COG0331 | COG0511 | 1 | 0 | 1 | 0 | 1 | 0 | 1 | 0 |
| COG0331 | COG0777 | 1 | 0 | 1 | 0 | 1 | 0 | 1 | 0 |
| COG0331 | COG0825 | 1 | 0 | 1 | 0 | 1 | 0 | 1 | 0 |
| COG0337 | COG0710 | 1 | 0 | 1 | 0 | 1 | 0 | 1 | 0 |
| COG0337 | COG0722 | 1 | 0 | 1 | 0 | 1 | 0 | 1 | 0 |
| COG0340 | COG0439 | 0 | 1 | 1 | 0 | 1 | 0 | 1 | 0 |
| COG0346 | COG0491 | 0 | 1 | 0 | 1 | 0 | 1 | 1 | 0 |
| COG0346 | COG1803 | 0 | 1 | 1 | 0 | 1 | 0 | 1 | 0 |
| COG0351 | COG0352 | 1 | 0 | 1 | 0 | 1 | 0 | 1 | 0 |

| | | | | | | | | | |
|---|---|---|---|---|---|---|---|---|---|
| COG0351 | COG0422 | 1 | 0 | 1 | 0 | 1 | 0 | 1 | 0 |
| COG0352 | COG0611 | 1 | 0 | 1 | 0 | 1 | 0 | 1 | 0 |
| COG0352 | COG1060 | 1 | 0 | 1 | 0 | 1 | 0 | 1 | 0 |
| COG0352 | COG2022 | 0 | 1 | 1 | 0 | 1 | 0 | 1 | 0 |
| COG0352 | COG2145 | 1 | 0 | 1 | 0 | 1 | 0 | 1 | 0 |
| COG0363 | COG1820 | 1 | 0 | 1 | 0 | 1 | 0 | 1 | 0 |
| COG0368 | COG2087 | 1 | 0 | 1 | 0 | 1 | 0 | 1 | 0 |
| COG0372 | COG1049 | 1 | 0 | 1 | 0 | 1 | 0 | 1 | 0 |
| COG0376 | COG0605 | 1 | 0 | 1 | 0 | 1 | 0 | 1 | 0 |
| COG0380 | COG1877 | 1 | 0 | 1 | 0 | 1 | 0 | 1 | 0 |
| COG0381 | COG0677 | 1 | 0 | 1 | 0 | 1 | 0 | 1 | 0 |
| COG0382 | COG3161 | 1 | 0 | 1 | 0 | 1 | 0 | 1 | 0 |
| COG0399 | COG0454 | 0 | 1 | 0 | 1 | 0 | 1 | 1 | 0 |
| COG0407 | COG0408 | 1 | 0 | 1 | 0 | 1 | 0 | 1 | 0 |
| COG0407 | COG0635 | 1 | 0 | 1 | 0 | 1 | 0 | 1 | 0 |
| COG0407 | COG1893 | 1 | 0 | 1 | 0 | 1 | 0 | 1 | 0 |
| COG0408 | COG0853 | 1 | 0 | 1 | 0 | 1 | 0 | 1 | 0 |
| COG0413 | COG1893 | 1 | 0 | 1 | 0 | 1 | 0 | 1 | 0 |
| COG0414 | COG0853 | 1 | 0 | 1 | 0 | 1 | 0 | 1 | 0 |
| COG0414 | COG1893 | 1 | 0 | 1 | 0 | 1 | 0 | 1 | 0 |
| COG0418 | COG0540 | 1 | 0 | 1 | 0 | 1 | 0 | 1 | 0 |
| COG0418 | COG1781 | 1 | 0 | 1 | 0 | 1 | 0 | 1 | 0 |
| COG0421 | COG1586 | 1 | 0 | 1 | 0 | 1 | 0 | 1 | 0 |
| COG0431 | COG2141 | 0 | 1 | 0 | 1 | 0 | 1 | 0 | 1 |
| COG0439 | COG1654 | 0 | 1 | 0 | 1 | 0 | 1 | 0 | 1 |
| COG0447 | COG1575 | 1 | 0 | 1 | 0 | 1 | 0 | 1 | 0 |
| COG0451 | COG1089 | 1 | 0 | 1 | 0 | 1 | 0 | 1 | 0 |
| COG0452 | COG0669 | 1 | 0 | 1 | 0 | 1 | 0 | 1 | 0 |
| COG0452 | COG1072 | 1 | 0 | 1 | 0 | 1 | 0 | 1 | 0 |
| COG0503 | COG0813 | 1 | 0 | 1 | 0 | 1 | 0 | 1 | 0 |
| COG0516 | COG0518 | 1 | 0 | 1 | 0 | 1 | 0 | 1 | 0 |
| COG0516 | COG0519 | 1 | 0 | 1 | 0 | 1 | 0 | 1 | 0 |
| COG0529 | COG2895 | 1 | 0 | 1 | 0 | 1 | 0 | 1 | 0 |
| COG0558 | COG0575 | 1 | 0 | 1 | 0 | 1 | 0 | 1 | 0 |
| COG0558 | COG0671 | 1 | 0 | 1 | 0 | 1 | 0 | 1 | 0 |
| COG0558 | COG1267 | 1 | 0 | 1 | 0 | 1 | 0 | 1 | 0 |
| COG0575 | COG1502 | 1 | 0 | 1 | 0 | 1 | 0 | 1 | 0 |
| COG0605 | COG0753 | 1 | 0 | 1 | 0 | 1 | 0 | 1 | 0 |
| COG0661 | COG2227 | 0 | 1 | 0 | 1 | 0 | 1 | 1 | 0 |
| COG0671 | COG1502 | 1 | 0 | 1 | 0 | 1 | 0 | 1 | 0 |
| COG0677 | COG1922 | 1 | 0 | 1 | 0 | 1 | 0 | 1 | 0 |
| COG0688 | COG1502 | 1 | 0 | 1 | 0 | 1 | 0 | 1 | 0 |
| COG0717 | COG0756 | 1 | 0 | 1 | 0 | 1 | 0 | 1 | 0 |
| COG0743 | COG1211 | 1 | 0 | 1 | 0 | 1 | 0 | 1 | 0 |
| COG0761 | COG0821 | 0 | 1 | 0 | 1 | 0 | 1 | 0 | 1 |
| COG0763 | COG1044 | 1 | 0 | 1 | 0 | 1 | 0 | 1 | 0 |
| COG0763 | COG2908 | 0 | 1 | 0 | 1 | 0 | 1 | 0 | 1 |
| COG0766 | COG0812 | 1 | 0 | 1 | 0 | 1 | 0 | 1 | 0 |
| COG0769 | COG0770 | 1 | 0 | 1 | 0 | 1 | 0 | 1 | 0 |
| COG0769 | COG0771 | 1 | 0 | 1 | 0 | 1 | 0 | 1 | 0 |
| COG0770 | COG1181 | 1 | 0 | 1 | 0 | 1 | 0 | 1 | 0 |
| COG0771 | COG0773 | 1 | 0 | 1 | 0 | 1 | 0 | 1 | 0 |
| COG0771 | COG0796 | 1 | 0 | 1 | 0 | 1 | 0 | 1 | 0 |
| COG0773 | COG0812 | 1 | 0 | 1 | 0 | 1 | 0 | 1 | 0 |
| COG0774 | COG1043 | 1 | 0 | 1 | 0 | 1 | 0 | 1 | 0 |
| COG0774 | COG1044 | 1 | 0 | 1 | 0 | 1 | 0 | 1 | 0 |

| | | | | | | | | | |
|---|---|---|---|---|---|---|---|---|---|
| COG0794 | COG2877 | 1 | 0 | 1 | 0 | 1 | 0 | 1 | 0 |
| COG0801 | COG1539 | 1 | 0 | 1 | 0 | 1 | 0 | 1 | 0 |
| COG0807 | COG1985 | 1 | 0 | 1 | 0 | 1 | 0 | 1 | 0 |
| COG0837 | COG1626 | 1 | 0 | 1 | 0 | 1 | 0 | 1 | 0 |
| COG0854 | COG1995 | 1 | 0 | 1 | 0 | 1 | 0 | 1 | 0 |
| COG1018 | COG1541 | 0 | 1 | 0 | 1 | 0 | 1 | 1 | 0 |
| COG1044 | COG2908 | 0 | 1 | 0 | 1 | 0 | 1 | 0 | 1 |
| COG1069 | COG2160 | 0 | 1 | 1 | 0 | 1 | 0 | 1 | 0 |
| COG1088 | COG1209 | 1 | 0 | 1 | 0 | 1 | 0 | 1 | 0 |
| COG1165 | COG1441 | 1 | 0 | 1 | 0 | 1 | 0 | 1 | 0 |
| COG1211 | COG1947 | 1 | 0 | 1 | 0 | 1 | 0 | 1 | 0 |
| COG1212 | COG1519 | 1 | 0 | 1 | 0 | 1 | 0 | 1 | 0 |
| COG1267 | COG1502 | 1 | 0 | 1 | 0 | 1 | 0 | 1 | 0 |
| COG1335 | COG1488 | 0 | 1 | 0 | 1 | 1 | 0 | 1 | 0 |
| COG1352 | COG2201 | 1 | 0 | 1 | 0 | 1 | 0 | 1 | 0 |
| COG1519 | COG1663 | 0 | 1 | 1 | 0 | 1 | 0 | 1 | 0 |
| COG1541 | COG2151 | 0 | 1 | 0 | 1 | 0 | 1 | 1 | 0 |
| COG1541 | COG3396 | 0 | 1 | 0 | 1 | 0 | 1 | 1 | 0 |
| COG1587 | COG1648 | 1 | 0 | 1 | 0 | 1 | 0 | 1 | 0 |
| COG1767 | COG3697 | 1 | 0 | 1 | 0 | 1 | 0 | 1 | 0 |
| COG1778 | COG2877 | 0 | 1 | 0 | 1 | 0 | 1 | 0 | 1 |
| COG1929 | COG2084 | 0 | 1 | 0 | 1 | 0 | 1 | 1 | 0 |
| COG1940 | COG3010 | 0 | 1 | 0 | 1 | 0 | 1 | 0 | 1 |
| COG2087 | COG2109 | 1 | 0 | 1 | 0 | 1 | 0 | 1 | 0 |
| COG3138 | COG3724 | 1 | 0 | 1 | 0 | 1 | 0 | 1 | 0 |
| | | 162 | 30 | 170 | 22 | 172 | 20 | 184 | 8 |
| | | 0.84 | 0.2 | 0.8854 | 0 | 0.8958 | 0 | 0.9583 | 0 |
