# Supplementary material for: Inferring modules of functionally interacting proteins using the Bond Energy Algorithm
Source: BMC Bioinformatics. 2008 Jun 17;9:285. doi: 10.1186/1471-2105-9-285 (PMC2474619; doi:10.1186/1471-2105-9-285)
Supplement: Additional file 3 — Result of BEA cluster. [file 1471-2105-9-285-S3.pdf]

name	cluster	funcion	clasificacion	valid yes	valid no
COG0470	1	L	J	1	0
COG0013	1	J	J	1	0
COG0621	1	J	J	1	0
COG1187	1	J	J	1	0
COG2813	1	J	J	1	0
COG1514	1	J	J	1	0
COG1670	1	J	J	1	0
COG3231	1	J	J	1	0
COG2511	1	J	J	1	0
COG0018	1	J	J	1	0
COG0154	1	J	J	1	0
COG0064	1	J	J	1	0
COG0721	1	J	J	1	0
COG0173	1	J	J	1	0
COG0017	1	J	J	1	0
COG0215	1	J	J	1	0
COG1490	1	J	J	1	0
COG1899	1	J	J	1	0
COG0030	1	J	J	1	0
COG1798	1	J	J	1	0
COG1736	1	J	J	1	0
COG1976	1	J	J	1	0
COG1889	1	J	J	1	0
COG0050	1	JE	J	1	0
COG0008	1	J	J	1	0
COG0752	1	J	J	1	0
COG0751	1	J	J	1	0
COG0423	1	J	J	1	0
COG0124	1	J	J	1	0
COG1431	1	J	J	1	0
COG0060	1	J	J	1	0
COG0495	1	J	J	1	0
COG1384	1	J	J	1	0
COG1190	1	J	J	1	0
COG0024	1	J	J	1	0
COG0223	1	J	J	1	0
COG0143	1	J	J	1	0
COG0293	1	J	J	1	0
COG0242	1	J	J	1	0
COG1867	1	J	J	1	0
COG1499	1	J	J	1	0
COG2016	1	J	J	1	0
COG1503	1	J	J	1	0
COG0193	1	J	J	1	0
COG0016	1	J	J	1	0

COG2024	1 J	J	1	0
COG0072	1 J	J	1	0
COG1185	1 J	J	1	0
COG2263	1 J	J	1	0
COG1491	1 J	J	1	0
COG1096	1 J	J	1	0
COG1534	1 J	J	1	0
COG2519	1 J	J	1	0
COG2260	1 J	J	1	0
COG2888	1 J	J	1	0
COG1236	1 J	J	1	0
COG1325	1 J	J	1	0
COG1500	1 J	J	1	0
COG1258	1 J	J	1	0
COG0219	1 J	J	1	0
COG1189	1 J	J	1	0
COG2890	1 J	J	1	0
COG2868	1 J	J	1	0
COG2117	1 J	J	1	0
COG0482	1 J	J	1	0
COG0442	1 J	J	1	0
COG0216	1 J	J	1	0
COG1186	1 J	J	1	0
COG2136	1 J	J	1	0
COG1498	1 J	J	1	0
COG1374	1 J	J	1	0
COG0130	1 J	J	1	0
COG0101	1 J	J	1	0
COG0564	1 J	J	1	0
COG0009	1 J	J	1	0
COG0251	1 J	J	1	0
COG1549	1 J	J	1	0
COG0343	1 J	J	1	0
COG1097	1 J	J	1	0
COG3277	1 J	J	1	0
COG1859	1 J	J	1	0
COG1588	1 J	J	1	0
COG2023	1 J	J	1	0
COG0594	1 J	J	1	0
COG1369	1 J	J	1	0
COG0689	1 J	J	1	0
COG2123	1 J	J	1	0
COG0349	1 J	J	1	0
COG3719	1 J	J	1	0
COG1603	1 J	J	1	0

COG1530	1 J	J	1	0
COG1358	1 J	J	1	0
COG0081	1 J	J	1	0
COG0244	1 J	J	1	0
COG0080	1 J	J	1	0
COG2264	1 J	J	1	0
COG2058	1 J	J	1	0
COG0102	1 J	J	1	0
COG0093	1 J	J	1	0
COG2163	1 J	J	1	0
COG0200	1 J	J	1	0
COG1632	1 J	J	1	0
COG0197	1 J	J	1	0
COG0203	1 J	J	1	0
COG0256	1 J	J	1	0
COG1727	1 J	J	1	0
COG0335	1 J	J	1	0
COG2147	1 J	J	1	0
COG0090	1 J	J	1	0
COG0292	1 J	J	1	0
COG2157	1 J	J	1	0
COG0261	1 J	J	1	0
COG2139	1 J	J	1	0
COG0091	1 J	J	1	0
COG0089	1 J	J	1	0
COG0198	1 J	J	1	0
COG2075	1 J	J	1	0
COG1825	1 J	J	1	0
COG0211	1 J	J	1	0
COG0227	1 J	J	1	0
COG0255	1 J	J	1	0
COG0087	1 J	J	1	0
COG1841	1 J	J	1	0
COG1911	1 J	J	1	0
COG0254	1 J	J	1	0
COG2097	1 J	J	1	0
COG0333	1 J	J	1	0
COG1717	1 J	J	1	0
COG0267	1 J	J	1	0
COG0230	1 J	J	1	0
COG2174	1 J	J	1	0
COG0291	1 J	J	1	0
COG2451	1 J	J	1	0
COG0257	1 J	J	1	0
COG1997	1 J	J	1	0

COG2126	1 J	J	1	0
COG2167	1 J	J	1	0
COG0088	1 J	J	1	0
COG1552	1 J	J	1	0
COG1631	1 J	J	1	0
COG0094	1 J	J	1	0
COG0097	1 J	J	1	0
COG0222	1 J	J	1	0
COG0359	1 J	J	1	0
COG0539	1 J	J	1	0
COG1098	1 J	J	1	0
COG0051	1 J	J	1	0
COG0100	1 J	J	1	0
COG0048	1 J	J	1	0
COG0099	1 J	J	1	0
COG0199	1 J	J	1	0
COG0184	1 J	J	1	0
COG0228	1 J	J	1	0
COG0186	1 J	J	1	0
COG1383	1 J	J	1	0
COG0238	1 J	J	1	0
COG0185	1 J	J	1	0
COG2238	1 J	J	1	0
COG0052	1 J	J	1	0
COG0268	1 J	J	1	0
COG0828	1 J	J	1	0
COG2004	1 J	J	1	0
COG1998	1 J	J	1	0
COG2051	1 J	J	1	0
COG2053	1 J	J	1	0
COG0092	1 J	J	1	0
COG1890	1 J	J	1	0
COG0522	1 J	J	1	0
COG1471	1 J	J	1	0
COG0098	1 J	J	1	0
COG0360	1 J	J	1	0
COG2125	1 J	J	1	0
COG0049	1 J	J	1	0
COG0096	1 J	J	1	0
COG2007	1 J	J	1	0
COG0103	1 J	J	1	0
COG3130	1 J	J	1	0
COG0233	1 J	J	1	0
COG1188	1 J	J	1	0
COG1544	1 J	J	1	0

COG0858	1 J	J	1	0
COG0806	1 J	J	1	0
COG0809	1 J	J	1	0
COG2265	1 J	J	1	0
COG3276	1 J	J	1	0
COG0172	1 J	J	1	0
COG2913	1 J	J	1	0
COG0441	1 J	J	1	0
COG0480	1 J	J	1	0
COG2092	1 J	J	1	0
COG0231	1 J	J	1	0
COG0264	1 J	J	1	0
COG0023	1 J	J	1	0
COG0532	1 J	J	1	0
COG0361	1 J	J	1	0
COG0290	1 J	J	1	0
COG1601	1 J	J	1	0
COG0182	1 J	J	1	0
COG1184	1 J	J	1	0
COG1093	1 J	J	1	0
COG2269	1 J	J	1	0
COG0180	1 J	J	1	0
COG0162	1 J	J	1	0
COG3557	1 J	J	1	0
COG0525	1 J	J	1	0
COG0565	1 J	J	1	0
COG0566	1 J	J	1	0
COG0144	1 J	J	1	0
COG0324	1 J	J	1	0
COG1746	1 J	J	1	0
COG0617	1 J	J	1	0
COG1676	1 J	J	1	0
COG0336	1 J	J	1	0
COG2909	2 K	K	1	0
COG2207	2 K	K	1	0
COG1581	2 K	K	1	0
COG2732	2 K	K	1	0
COG1654	2 KH	K	1	0
COG1278	2 K	K	1	0
COG2771	2 K	K	1	0
COG3710	2 K	K	1	0
COG0202	2 K	K	1	0
COG0085	2 K	K	1	0
COG0086	2 K	K	1	0
COG3343	2 K	K	1	0

COG0568	2 K	K	1	0
COG1191	2 K	K	1	0
COG1595	2 K	K	1	0
COG1508	2 K	K	1	0
COG1095	2 K	K	1	0
COG2093	2 K	K	1	0
COG1758	2 K	K	1	0
COG1594	2 K	K	1	0
COG1996	2 K	K	1	0
COG2012	2 K	K	1	0
COG1761	2 K	K	1	0
COG1644	2 K	K	1	0
COG1243	2 K	K	1	0
COG0557	2 K	K	1	0
COG2901	2 KL	K	1	0
COG1954	2 K	K	1	0
COG0454	2 KR	K	1	0
COG1321	2 K	K	1	0
COG2747	2 KN	K	1	0
COG3561	2 K	K	1	0
COG3327	2 K	K	1	0
COG1293	2 K	K	1	0
COG2740	2 K	K	1	0
COG1813	2 K	K	1	0
COG1959	2 K	K	1	0
COG2345	2 K	K	1	0
COG2378	2 K	K	1	0
COG2462	2 K	K	1	0
COG2932	2 K	K	1	0
COG2944	2 K	K	1	0
COG3311	2 K	K	1	0
COG3355	2 K	K	1	0
COG3423	2 K	K	1	0
COG3432	2 K	K	1	0
COG3636	2 K	K	1	0
COG3655	2 K	K	1	0
COG3682	2 K	K	1	0
COG2865	2 K	K	1	0
COG3357	2 K	K	1	0
COG1386	2 K	K	1	0
COG2524	2 K	K	1	0
COG3620	2 K	K	1	0
COG1327	2 K	K	1	0
COG0640	2 K	K	1	0
COG0789	2 K	K	1	0

COG1318	2	K	K	1	0
COG1378	2	K	K	1	0
COG1395	2	K	K	1	0
COG1396	2	K	K	1	0
COG1475	2	K	K	1	0
COG1476	2	K	K	1	0
COG1497	2	K	K	1	0
COG1510	2	K	K	1	0
COG1695	2	K	K	1	0
COG1709	2	K	K	1	0
COG1725	2	K	K	1	0
COG1733	2	K	K	1	0
COG1777	2	K	K	1	0
COG3609	2	K	K	1	0
COG0864	2	K	K	1	0
COG3617	2	K	K	1	0
COG1983	2	KT	K	1	0
COG0819	2	K	K	1	0
COG1678	2	K	K	1	0
COG1521	2	K	K	1	0
COG0430	2	K	K	1	0
COG0724	2	K	K	1	0
COG3160	2	K	K	1	0
COG2002	2	K	K	1	0
COG3437	2	KT	K	1	0
COG3279	2	KT	K	1	0
COG1974	2	KT	K	1	0
COG1958	2	K	K	1	0
COG3835	2	KT	K	1	0
COG0553	2	KL	K	1	0
COG0250	2	K	K	1	0
COG0195	2	K	K	1	0
COG0782	2	K	K	1	0
COG1308	2	K	K	1	0
COG1405	2	K	K	1	0
COG1675	2	K	K	1	0
COG2101	2	K	K	1	0
COG0781	2	K	K	1	0
COG1158	2	K	K	1	0
COG3711	2	K	K	1	0
COG0583	2	K	K	1	0
COG1309	2	K	K	1	0
COG1316	2	K	K	1	0
COG1414	2	K	K	1	0
COG2808	2	K	K	1	0

COG3722	2 K	K	1	0
COG3604	2 KT	K	1	0
COG3829	2 KT	K	1	0
COG1339	2 K	K	1	0
COG3283	2 K	K	1	0
COG1420	2 K	K	1	0
COG2390	2 K	K	1	0
COG1522	2 K	K	1	0
COG1609	2 K	K	1	0
COG1737	2 K	K	1	0
COG1802	2 K	K	1	0
COG1846	2 K	K	1	0
COG1940	2 K	K	1	0
COG2186	2 K	K	1	0
COG2188	2 K	K	1	0
COG1167	2 KE	K	1	0
COG1349	2 KG	K	1	0
COG1329	2 K	K	1	0
COG2973	2 K	K	1	0
COG0571	2 K	K	1	0
COG0122	3 L	L	1	0
COG2818	3 L	L	1	0
COG2094	3 L	L	1	0
COG0258	3 L	L	1	0
COG1194	3 L	L	1	0
COG1793	3 L	L	1	0
COG1423	3 L	L	1	0
COG1074	3 L	L	1	0
COG0507	3 L	L	1	0
COG0419	3 L	L	1	0
COG0593	3 L	L	1	0
COG0497	3 L	L	1	0
COG2189	3 L	L	1	0
COG0827	3 L	L	1	0
COG3392	3 L	L	1	0
COG3145	3 L	L	1	0
COG1107	3 L	L	1	0
COG0776	3 L	L	1	0
COG1474	3 LO	L	1	0
COG3727	3 L	L	1	0
COG0188	3 L	L	1	0
COG0187	3 L	L	1	0
COG3449	3 L	L	1	0
COG1224	3 L	L	1	0
COG0323	3 L	L	1	0

COG3066	3 L	L	1	0
COG0863	3 L	L	1	0
COG1061	3 L	L	1	0
COG0749	3 L	L	1	0
COG1311	3 L	L	1	0
COG0587	3 L	L	1	0
COG2176	3 L	L	1	0
COG2927	3 L	L	1	0
COG1466	3 L	L	1	0
COG0847	3 L	L	1	0
COG2812	3 L	L	1	0
COG3050	3 L	L	1	0
COG1796	3 L	L	1	0
COG0417	3 L	L	1	0
COG0592	3 L	L	1	0
COG0358	3 L	L	1	0
COG2974	3 L	L	1	0
COG0420	3 L	L	1	0
COG1533	3 L	L	1	0
COG2003	3 L	L	1	0
COG1484	3 L	L	1	0
COG1697	3 L	L	1	0
COG1389	3 L	L	1	0
COG1555	3 L	L	1	0
COG3077	3 L	L	1	0
COG1515	3 L	L	1	0
COG0415	3 L	L	1	0
COG1111	3 L	L	1	0
COG1948	3 L	L	1	0
COG2356	3 L	L	1	0
COG2231	3 L	L	1	0
COG0648	3 L	L	1	0
COG2219	3 L	L	1	0
COG1467	3 L	L	1	0
COG0178	3 L	L	1	0
COG2925	3 L	L	1	0
COG0708	3 L	L	1	0
COG1330	3 L	L	1	0
COG1722	3 L	L	1	0
COG1570	3 L	L	1	0
COG0266	3 L	L	1	0
COG3663	3 L	L	1	0
COG1401	3 L	L	1	0
COG0556	3 L	L	1	0
COG2036	3 L	L	1	0

COG1591	3	L	L	1	0
COG0632	3	L	L	1	0
COG0817	3	L	L	1	0
COG2255	3	L	L	1	0
COG1643	3	L	L	1	0
COG3039	3	L	L	1	0
COG1662	3	L	L	1	0
COG0582	3	L	L	1	0
COG1372	3	L	L	1	0
COG3780	3	L	L	1	0
COG0350	3	L	L	1	0
COG0084	3	L	L	1	0
COG1525	3	L	L	1	0
COG1193	3	L	L	1	0
COG0249	3	L	L	1	0
COG0742	3	L	L	1	0
COG1933	3	L	L	1	0
COG0272	3	L	L	1	0
COG2816	3	L	L	1	0
COG0494	3	LR	L	1	0
COG3057	3	L	L	1	0
COG0322	3	L	L	1	0
COG0389	3	L	L	1	0
COG3728	3	L	L	1	0
COG3298	3	L	L	1	0
COG3593	3	L	L	1	0
COG1241	3	L	L	1	0
COG1743	3	L	L	1	0
COG1041	3	L	L	1	0
COG3569	3	L	L	1	0
COG0177	3	L	L	1	0
COG2254	3	L	L	1	0
COG0116	3	L	L	1	0
COG0758	3	LN	L	1	0
COG2827	3	L	L	1	0
COG0792	3	L	L	1	0
COG1787	3	L	L	1	0
COG0816	3	L	L	1	0
COG3285	3	L	L	1	0
COG3359	3	L	L	1	0
COG3695	3	L	L	1	0
COG1637	3	L	L	1	0
COG3183	3	L	L	1	0
COG3440	3	L	L	1	0
COG2452	3	L	L	1	0

COG1425	3 L	L	1	0
COG1943	3 L	L	1	0
COG3293	3 L	L	1	0
COG3328	3 L	L	1	0
COG3335	3 L	L	1	0
COG3385	3 L	L	1	0
COG0675	3 L	L	1	0
COG2810	3 L	L	1	0
COG1198	3 L	L	1	0
COG2965	3 L	L	1	0
COG2946	3 L	L	1	0
COG2801	3 L	L	1	0
COG3316	3 L	L	1	0
COG3415	3 L	L	1	0
COG1199	3 L	L	1	0
COG3598	3 L	L	1	0
COG0468	3 L	L	1	0
COG1468	3 L	L	1	0
COG2887	3 L	L	1	0
COG1200	3 LK	L	1	0
COG1195	3 L	L	1	0
COG3723	3 L	L	1	0
COG0353	3 L	L	1	0
COG1381	3 L	L	1	0
COG1599	3 L	L	1	0
COG3611	3 L	L	1	0
COG0305	3 L	L	1	0
COG1403	3 L	L	1	0
COG1715	3 L	L	1	0
COG3587	3 L	L	1	0
COG0732	3 L	L	1	0
COG0610	3 L	L	1	0
COG3344	3 L	L	1	0
COG1110	3 L	L	1	0
COG0328	3 L	L	1	0
COG0164	3 L	L	1	0
COG1039	3 L	L	1	0
COG0629	3 L	L	1	0
COG0608	3 L	L	1	0
COG0270	3 L	L	1	0
COG0338	3 L	L	1	0
COG1961	3 L	L	1	0
COG1658	3 L	L	1	0
COG0783	3 L	L	1	0
COG0210	3 L	L	1	0

COG1112	3	L	L	1	0
COG0513	3	LKJ	L	1	0
COG0514	3	L	L	1	0
COG1059	3	L	L	1	0
COG0550	3	L	L	1	0
COG1197	3	LK	L	1	0
COG2178	3	L	L	1	0
COG2963	3	L	L	1	0
COG3436	3	L	L	1	0
COG3464	3	L	L	1	0
COG3547	3	L	L	1	0
COG3666	3	L	L	1	0
COG3676	3	L	L	1	0
COG3677	3	L	L	1	0
COG2826	3	L	L	1	0
COG0286	3	L	L	1	0
COG1002	3	L	L	1	0
COG2256	3	L	L	1	0
COG0692	3	L	L	1	0
COG1573	3	L	L	1	0
COG0551	3	L	L	1	0
COG0455	4	D	D	1	0
COG0489	4	D	D	1	0
COG1192	4	D	D	1	0
COG0772	4	D	D	1	0
COG3640	4	D	D	1	0
COG0206	4	D	D	1	0
COG3599	4	D	D	1	0
COG2177	4	D	D	1	0
COG3087	4	D	D	1	0
COG3115	4	D	D	1	0
COG3116	4	D	D	1	0
COG1196	4	D	D	1	0
COG1674	4	D	D	1	0
COG1077	4	D	D	1	0
COG0239	4	D	D	1	0
COG2917	4	D	D	1	0
COG1206	4	D	D	1	0
COG0445	4	D	D	1	0
COG0424	4	D	D	1	0
COG2884	4	D	D	1	0
COG0003	4	D	D	1	0
COG0037	4	D	D	1	0
COG0849	4	D	D	1	0
COG2184	4	D	D	1	0

COG2846	4	D	D	1	0
COG0850	4	D	D	1	0
COG2894	4	D	D	1	0
COG0851	4	D	D	1	0
COG2385	4	D	D	1	0
COG3006	4	D	D	1	0
COG3095	4	D	D	1	0
COG3096	4	D	D	1	0
COG0755	5	O	O	1	0
COG2386	5	O	O	1	0
COG2994	5	O	O	1	0
COG1397	5	O	O	1	0
COG1222	5	O	O	1	0
COG0466	5	O	O	1	0
COG0465	5	O	O	1	0
COG1219	5	O	O	1	0
COG1220	5	O	O	1	0
COG0464	5	O	O	1	0
COG0542	5	O	O	1	0
COG3634	5	O	O	1	0
COG3555	5	O	O	1	0
COG0459	5	O	O	1	0
COG0234	5	O	O	1	0
COG0826	5	O	O	1	0
COG0229	5	O	O	1	0
COG1138	5	O	O	1	0
COG0785	5	O	O	1	0
COG2332	5	O	O	1	0
COG1281	5	O	O	1	0
COG1495	5	O	O	1	0
COG1076	5	O	O	1	0
COG1928	5	O	O	1	0
COG0544	5	O	O	1	0
COG0545	5	O	O	1	0
COG1047	5	O	O	1	0
COG3823	5	O	O	1	0
COG1391	5	OT	O	1	0
COG2999	5	O	O	1	0
COG0695	5	O	O	1	0
COG0278	5	O	O	1	0
COG0386	5	O	O	1	0
COG0625	5	O	O	1	0
COG3187	5	O	O	1	0
COG0068	5	O	O	1	0
COG0298	5	O	O	1	0

COG0309	5	O	O	1	0
COG0409	5	O	O	1	0
COG1973	5	O	O	1	0
COG2370	5	O	O	1	0
COG1214	5	O	O	1	0
COG2360	5	O	O	1	0
COG0330	5	O	O	1	0
COG0533	5	O	O	1	0
COG0443	5	O	O	1	0
COG0071	5	O	O	1	0
COG0576	5	O	O	1	0
COG0326	5	O	O	1	0
COG0484	5	O	O	1	0
COG2214	5	O	O	1	0
COG0378	5	OK	O	1	0
COG0602	5	O	O	1	0
COG0760	5	O	O	1	0
COG0225	5	O	O	1	0
COG0652	5	O	O	1	0
COG0450	5	O	O	1	0
COG0678	5	O	O	1	0
COG1225	5	O	O	1	0
COG2077	5	O	O	1	0
COG1067	5	O	O	1	0
COG1066	5	O	O	1	0
COG0606	5	O	O	1	0
COG1223	5	O	O	1	0
COG2192	5	O	O	1	0
COG0435	5	O	O	1	0
COG3590	5	O	O	1	0
COG1730	5	O	O	1	0
COG3751	5	O	O	1	0
COG3484	5	O	O	1	0
COG3531	5	O	O	1	0
COG0425	5	O	O	1	0
COG1764	5	O	O	1	0
COG1765	5	O	O	1	0
COG1382	5	O	O	1	0
COG1370	5	O	O	1	0
COG0638	5	O	O	1	0
COG2518	5	O	O	1	0
COG1651	5	O	O	1	0
COG2935	5	O	O	1	0
COG2020	5	O	O	1	0
COG2039	5	O	O	1	0

COG1180	5	O	O	1	0
COG1333	5	O	O	1	0
COG1025	5	O	O	1	0
COG2919	5	O	O	1	0
COG1404	5	O	O	1	0
COG0526	5	OC	O	1	0
COG3118	5	O	O	1	0
COG0492	5	O	O	1	0
COG0694	5	O	O	1	0
COG2143	5	O	O	1	0
COG0265	5	O	O	1	0
COG2844	5	O	O	1	0
COG3526	5	O	O	1	0
COG3088	5	O	O	1	0
COG3058	5	O	O	1	0
COG1612	5	O	O	1	0
COG2371	5	O	O	1	0
COG0830	5	O	O	1	0
COG0829	5	O	O	1	0
COG1975	5	O	O	1	0
COG0501	5	O	O	1	0
COG0691	5	O	O	1	0
COG2877	6	M	M	1	0
COG1519	6	M	M	1	0
COG1807	6	M	M	1	0
COG1682	6	M	M	1	0
COG2870	6	M	M	1	0
COG0859	6	M	M	1	0
COG1043	6	M	M	1	0
COG0787	6	M	M	1	0
COG0815	6	M	M	1	0
COG1794	6	M	M	1	0
COG3040	6	M	M	1	0
COG2367	6	M	M	1	0
COG1680	6	M	M	1	0
COG2602	6	M	M	1	0
COG1212	6	M	M	1	0
COG1083	6	M	M	1	0
COG3524	6	M	M	1	0
COG3562	6	M	M	1	0
COG3563	6	M	M	1	0
COG3659	6	M	M	1	0
COG0768	6	M	M	1	0
COG1589	6	M	M	1	0
COG3061	6	M	M	1	0

COG3773	6	M	M	1	0
COG0791	6	M	M	1	0
COG3765	6	M	M	1	0
COG1292	6	M	M	1	0
COG2230	6	M	M	1	0
COG0615	6	MI	M	1	0
COG1181	6	M	M	1	0
COG1686	6	M	M	1	0
COG1876	6	M	M	1	0
COG2027	6	M	M	1	0
COG2173	6	M	M	1	0
COG0818	6	M	M	1	0
COG0821	6	M	M	1	0
COG1089	6	M	M	1	0
COG0449	6	M	M	1	0
COG0796	6	M	M	1	0
COG3306	6	M	M	1	0
COG0463	6	M	M	1	0
COG1215	6	M	M	1	0
COG3475	6	M	M	1	0
COG1970	6	M	M	1	0
COG3307	6	M	M	1	0
COG0763	6	M	M	1	0
COG3754	6	M	M	1	0
COG1442	6	M	M	1	0
COG0797	6	M	M	1	0
COG3757	6	M	M	1	0
COG0836	6	M	M	1	0
COG3774	6	M	M	1	0
COG0744	6	M	M	1	0
COG2943	6	M	M	1	0
COG3064	6	M	M	1	0
COG0739	6	M	M	1	0
COG2821	6	M	M	1	0
COG2951	6	M	M	1	0
COG3770	6	M	M	1	0
COG1207	6	M	M	1	0
COG0860	6	M	M	1	0
COG3023	6	M	M	1	0
COG3248	6	M	M	1	0
COG0451	6	MG	M	1	0
COG1208	6	MJ	M	1	0
COG3637	6	M	M	1	0
COG1452	6	M	M	1	0
COG3133	6	M	M	1	0

COG3017	6 M	M	1	0
COG2834	6 M	M	1	0
COG2829	6 M	M	1	0
COG1538	6 MN	M	1	0
COG2825	6 M	M	1	0
COG3203	6 M	M	1	0
COG3713	6 M	M	1	0
COG3047	6 M	M	1	0
COG2885	6 M	M	1	0
COG3049	6 M	M	1	0
COG1732	6 M	M	1	0
COG0793	6 M	M	1	0
COG0810	6 M	M	1	0
COG1596	6 M	M	1	0
COG1368	6 M	M	1	0
COG3511	6 M	M	1	0
COG0357	6 M	M	1	0
COG0275	6 M	M	1	0
COG1004	6 M	M	1	0
COG0510	6 M	M	1	0
COG0438	6 M	M	1	0
COG1696	6 M	M	1	0
COG0750	6 M	M	1	0
COG1086	6 MG	M	1	0
COG3660	6 M	M	1	0
COG0702	6 MG	M	1	0
COG0729	6 M	M	1	0
COG3468	6 M	M	1	0
COG3699	6 M	M	1	0
COG2222	6 M	M	1	0
COG0399	6 M	M	1	0
COG1213	6 M	M	1	0
COG0794	6 M	M	1	0
COG0682	6 M	M	1	0
COG2247	6 M	M	1	0
COG1346	6 M	M	1	0
COG2843	6 M	M	1	0
COG3559	6 M	M	1	0
COG1887	6 M	M	1	0
COG3210	6 M	M	1	0
COG3409	6 M	M	1	0
COG3137	6 M	M	1	0
COG2980	6 M	M	1	0
COG3209	6 M	M	1	0
COG1792	6 M	M	1	0

COG2891	6 M	M	1	0
COG1361	6 M	M	1	0
COG2335	6 M	M	1	0
COG2089	6 M	M	1	0
COG0668	6 M	M	1	0
COG3264	6 M	M	1	0
COG0741	6 M	M	1	0
COG3764	6 M	M	1	0
COG1247	6 M	M	1	0
COG1861	6 M	M	1	0
COG3065	6 M	M	1	0
COG2148	6 M	M	1	0
COG2853	6 M	M	1	0
COG1922	6 M	M	1	0
COG1044	6 M	M	1	0
COG0774	6 M	M	1	0
COG0677	6 M	M	1	0
COG0381	6 M	M	1	0
COG0766	6 M	M	1	0
COG0707	6 M	M	1	0
COG0812	6 M	M	1	0
COG0773	6 M	M	1	0
COG0771	6 M	M	1	0
COG0472	6 M	M	1	0
COG0770	6 M	M	1	0
COG0769	6 M	M	1	0
COG0562	6 M	M	1	0
COG1087	6 M	M	1	0
COG1210	6 M	M	1	0
COG3056	6 M	M	1	0
COG3317	6 M	M	1	0
COG3015	6 MP	M	1	0
COG3170	6 M	M	1	0
COG1462	6 M	M	1	0
COG2982	6 M	M	1	0
COG2088	6 M	M	1	0
COG1898	6 M	M	1	0
COG1091	6 M	M	1	0
COG1088	6 M	M	1	0
COG1209	6 M	M	1	0
COG1127	7 N	N	1	0
COG2854	7 N	N	1	0
COG3839	7 N	N	1	0
COG1681	7 N	N	1	0
COG0848	7 N	N	1	0

COG0811	7 N	N	1	0
COG3143	7 N	N	1	0
COG1776	7 NT	N	1	0
COG0643	7 N	N	1	0
COG1871	7 NT	N	1	0
COG2201	7 NT	N	1	0
COG0835	7 N	N	1	0
COG2303	7 N	N	1	0
COG3166	7 N	N	1	0
COG3167	7 N	N	1	0
COG3168	7 N	N	1	0
COG1419	7 N	N	1	0
COG2063	7 N	N	1	0
COG1261	7 N	N	1	0
COG1749	7 N	N	1	0
COG1815	7 N	N	1	0
COG1558	7 N	N	1	0
COG1580	7 N	N	1	0
COG1706	7 N	N	1	0
COG3190	7 N	N	1	0
COG2882	7 N	N	1	0
COG1157	7 N	N	1	0
COG3418	7 N	N	1	0
COG1766	7 N	N	1	0
COG1298	7 N	N	1	0
COG1317	7 N	N	1	0
COG1338	7 N	N	1	0
COG1377	7 N	N	1	0
COG1684	7 N	N	1	0
COG1987	7 N	N	1	0
COG1345	7 N	N	1	0
COG1843	7 N	N	1	0
COG1256	7 N	N	1	0
COG1677	7 N	N	1	0
COG3144	7 N	N	1	0
COG1291	7 N	N	1	0
COG1360	7 N	N	1	0
COG1536	7 N	N	1	0
COG1868	7 N	N	1	0
COG1886	7 N	N	1	0
COG1516	7 N	N	1	0
COG1344	7 N	N	1	0
COG1705	7 N	N	1	0
COG3267	7 N	N	1	0
COG3031	7 N	N	1	0

COG3156	7 N	N	1	0
COG3149	7 N	N	1	0
COG1450	7 N	N	1	0
COG1459	7 N	N	1	0
COG3297	7 N	N	1	0
COG2165	7 N	N	1	0
COG3114	7 N	N	1	0
COG2831	7 N	N	1	0
COG1253	7 N	N	1	0
COG1560	7 N	N	1	0
COG1663	7 N	N	1	0
COG0597	7 N	N	1	0
COG1388	7 N	N	1	0
COG0481	7 N	N	1	0
COG1585	7 NO	N	1	0
COG1030	7 NO	N	1	0
COG0840	7 N	N	1	0
COG1352	7 NT	N	1	0
COG3188	7 N	N	1	0
COG3121	7 N	N	1	0
COG0823	7 N	N	1	0
COG0616	7 NO	N	1	0
COG3539	7 N	N	1	0
COG3847	7 N	N	1	0
COG3419	7 N	N	1	0
COG3093	7 N	N	1	0
COG3549	7 N	N	1	0
COG3668	7 N	N	1	0
COG2874	7 N	N	1	0
COG2804	7 N	N	1	0
COG2805	7 N	N	1	0
COG1406	7 N	N	1	0
COG1217	7 N	N	1	0
COG3417	7 N	N	1	0
COG3489	7 N	N	1	0
COG1724	7 N	N	1	0
COG2503	7 N	N	1	0
COG1314	7 N	N	1	0
COG0653	7 N	N	1	0
COG1952	7 N	N	1	0
COG0342	7 N	N	1	0
COG0690	7 N	N	1	0
COG0341	7 N	N	1	0
COG0201	7 N	N	1	0
COG1862	7 N	N	1	0

COG0706	7 N	N	1	0
COG3654	7 N	N	1	0
COG0740	7 NO	N	1	0
COG2443	7 N	N	1	0
COG3352	7 N	N	1	0
COG3351	7 N	N	1	0
COG3353	7 N	N	1	0
COG3354	7 N	N	1	0
COG3070	7 N	N	1	0
COG0805	7 N	N	1	0
COG1826	7 N	N	1	0
COG0681	7 N	N	1	0
COG1989	7 N	N	1	0
COG1400	7 N	N	1	0
COG0541	7 N	N	1	0
COG0552	7 N	N	1	0
COG1744	7 N	N	1	0
COG3215	7 N	N	1	0
COG3736	7 N	N	1	0
COG3701	7 N	N	1	0
COG3846	7 N	N	1	0
COG2948	7 N	N	1	0
COG0630	7 N	N	1	0
COG3838	7 N	N	1	0
COG3451	7 N	N	1	0
COG3704	7 N	N	1	0
COG3504	7 N	N	1	0
COG3843	7 N	N	1	0
COG3505	7 N	N	1	0
COG1334	7 N	N	1	0
COG1955	7 N	N	1	0
COG3206	7 N	N	1	0
COG3063	7 N	N	1	0
COG3745	7 N	N	1	0
COG1582	7 N	N	1	0
COG1218	8 P	P	1	0
COG0803	8 P	P	1	0
COG1121	8 P	P	1	0
COG1108	8 P	P	1	0
COG1120	8 PH	P	1	0
COG0614	8 P	P	1	0
COG0609	8 PH	P	1	0
COG1122	8 P	P	1	0
COG1930	8 P	P	1	0
COG0619	8 P	P	1	0

COG3841	8	P	P	1	0
COG0725	8	P	P	1	0
COG1119	8	P	P	1	0
COG1116	8	P	P	1	0
COG0715	8	P	P	1	0
COG0600	8	P	P	1	0
COG1117	8	P	P	1	0
COG0226	8	P	P	1	0
COG0573	8	P	P	1	0
COG0581	8	P	P	1	0
COG3638	8	P	P	1	0
COG3639	8	P	P	1	0
COG1613	8	P	P	1	0
COG1118	8	P	P	1	0
COG0555	8	P	P	1	0
COG2046	8	P	P	1	0
COG0529	8	P	P	1	0
COG1785	8	P	P	1	0
COG0004	8	P	P	1	0
COG1393	8	P	P	1	0
COG0798	8	P	P	1	0
COG3119	8	P	P	1	0
COG2193	8	P	P	1	0
COG2906	8	P	P	1	0
COG0387	8	P	P	1	0
COG0530	8	P	P	1	0
COG0288	8	P	P	1	0
COG3338	8	P	P	1	0
COG0753	8	P	P	1	0
COG0376	8	P	P	1	0
COG0474	8	P	P	1	0
COG2217	8	P	P	1	0
COG0038	8	P	P	1	0
COG2059	8	P	P	1	0
COG1230	8	P	P	1	0
COG2608	8	P	P	1	0
COG2032	8	P	P	1	0
COG1513	8	P	P	1	0
COG2807	8	P	P	1	0
COG1858	8	P	P	1	0
COG0471	8	P	P	1	0
COG2382	8	P	P	1	0
COG0370	8	P	P	1	0
COG1918	8	P	P	1	0
COG3712	8	PT	P	1	0

COG0735	8	P	P	1	0
COG2146	8	PR	P	1	0
COG1528	8	P	P	1	0
COG3301	8	P	P	1	0
COG3303	8	P	P	1	0
COG2116	8	P	P	1	0
COG2895	8	P	P	1	0
COG3265	8	P	P	1	0
COG3230	8	P	P	1	0
COG2703	8	P	P	1	0
COG0672	8	P	P	1	0
COG2216	8	P	P	1	0
COG3376	8	P	P	1	0
COG0569	8	P	P	1	0
COG3158	8	P	P	1	0
COG2060	8	P	P	1	0
COG2156	8	P	P	1	0
COG0475	8	P	P	1	0
COG1226	8	P	P	1	0
COG0861	8	P	P	1	0
COG2076	8	P	P	1	0
COG3454	8	P	P	1	0
COG2239	8	P	P	1	0
COG0598	8	P	P	1	0
COG3546	8	P	P	1	0
COG1914	8	P	P	1	0
COG1006	8	P	P	1	0
COG1320	8	P	P	1	0
COG1863	8	P	P	1	0
COG2111	8	P	P	1	0
COG2212	8	P	P	1	0
COG3004	8	P	P	1	0
COG3067	8	P	P	1	0
COG1055	8	P	P	1	0
COG1283	8	P	P	1	0
COG0025	8	P	P	1	0
COG1140	8	P	P	1	0
COG2223	8	P	P	1	0
COG3256	8	P	P	1	0
COG1348	8	P	P	1	0
COG3420	8	P	P	1	0
COG1629	8	P	P	1	0
COG3131	8	P	P	1	0
COG1910	8	P	P	1	0
COG1824	8	P	P	1	0

COG1392	8 P	P	1	0
COG0704	8 P	P	1	0
COG3746	8 P	P	1	0
COG0306	8 P	P	1	0
COG3540	8 P	P	1	0
COG0855	8 P	P	1	0
COG0053	8 P	P	1	0
COG0428	8 P	P	1	0
COG2072	8 P	P	1	0
COG2837	8 P	P	1	0
COG2822	8 P	P	1	0
COG1563	8 P	P	1	0
COG1965	8 P	P	1	0
COG1276	8 P	P	1	0
COG3720	8 P	P	1	0
COG0748	8 P	P	1	0
COG3721	8 P	P	1	0
COG3221	8 P	P	1	0
COG0490	8 P	P	1	0
COG3696	8 P	P	1	0
COG0607	8 P	P	1	0
COG2897	8 P	P	1	0
COG2375	8 P	P	1	0
COG0659	8 P	P	1	0
COG0369	8 P	P	1	0
COG0155	8 P	P	1	0
COG2920	8 P	P	1	0
COG0605	8 P	P	1	0
COG3793	8 P	P	1	0
COG1275	8 P	P	1	0
COG0168	8 P	P	1	0
COG1553	8 P	P	1	0
COG2168	8 P	P	1	0
COG3470	8 P	P	1	0
COG2824	8 P	P	1	0
COG3709	8 P	P	1	0
COG3624	8 P	P	1	0
COG3625	8 P	P	1	0
COG3626	8 P	P	1	0
COG3627	8 P	P	1	0
COG3487	8 P	P	1	0
COG2967	8 P	P	1	0
COG3703	8 P	P	1	0
COG3142	8 P	P	1	0
COG3667	8 P	P	1	0

COG3062	8 P	P	1	0
COG3213	8 P	P	1	0
COG2923	8 P	P	1	0
COG1324	8 P	P	1	0
COG3830	9 T	T	1	0
COG3109	9 T	T	1	0
COG2114	9 T	T	1	0
COG1366	9 T	T	1	0
COG3806	9 T	T	1	0
COG2172	9 T	T	1	0
COG3448	9 T	T	1	0
COG1966	9 T	T	1	0
COG1551	9 T	T	1	0
COG0784	9 T	T	1	0
COG3629	9 T	T	1	0
COG0123	9 TQ	T	1	0
COG0639	9 T	T	1	0
COG1734	9 T	T	1	0
COG2200	9 T	T	1	0
COG3456	9 T	T	1	0
COG1716	9 T	T	1	0
COG2203	9 T	T	1	0
COG1956	9 T	T	1	0
COG2199	9 T	T	1	0
COG2337	9 T	T	1	0
COG2336	9 T	T	1	0
COG0317	9 TK	T	1	0
COG2770	9 T	T	1	0
COG2206	9 T	T	1	0
COG2198	9 T	T	1	0
COG1854	9 T	T	1	0
COG3725	9 T	T	1	0
COG3642	9 T	T	1	0
COG3026	9 T	T	1	0
COG3073	9 T	T	1	0
COG2205	9 T	T	1	0
COG2202	9 T	T	1	0
COG3438	9 T	T	1	0
COG1702	9 T	T	1	0
COG2062	9 T	T	1	0
COG3848	9 T	T	1	0
COG3086	9 T	T	1	0
COG1875	9 T	T	1	0
COG2112	9 T	T	1	0
COG3300	9 T	T	1	0

COG3447	9	T	T	1	0
COG3292	9	T	T	1	0
COG3322	9	T	T	1	0
COG3452	9	T	T	1	0
COG3614	9	T	T	1	0
COG2453	9	T	T	1	0
COG3480	9	T	T	1	0
COG1718	9	T	T	1	0
COG1639	9	T	T	1	0
COG3434	9	T	T	1	0
COG2972	9	T	T	1	0
COG2905	9	T	T	1	0
COG0631	9	T	T	1	0
COG2365	9	T	T	1	0
COG0394	9	T	T	1	0
COG2766	9	T	T	1	0
COG3275	9	T	T	1	0
COG0478	9	T	T	1	0
COG0467	9	T	T	1	0
COG2508	9	TQ	T	1	0
COG2204	9	T	T	1	0
COG3706	9	T	T	1	0
COG2197	9	TK	T	1	0
COG3707	9	T	T	1	0
COG0745	9	TK	T	1	0
COG3103	9	T	T	1	0
COG1493	9	T	T	1	0
COG2208	9	TK	T	1	0
COG0515	9	T	T	1	0
COG0642	9	T	T	1	0
COG3290	9	T	T	1	0
COG3851	9	T	T	1	0
COG3850	9	T	T	1	0
COG3852	9	T	T	1	0
COG3605	9	T	T	1	0
COG0271	9	T	T	1	0
COG1221	9	TK	T	1	0
COG3476	9	T	T	1	0
COG3726	9	T	T	1	0
COG2310	9	T	T	1	0
COG0589	9	T	T	1	0
COG0664	9	T	T	1	0
COG1151	10	C	C	1	0
COG1668	10	C	C	1	0
COG3202	10	C	C	1	0

COG0282	10	C	C	1	0
COG0427	10	C	C	1	0
COG1048	10	C	C	1	0
COG1049	10	C	C	1	0
COG1042	10	C	C	1	0
COG1254	10	C	C	1	0
COG1529	10	C	C	1	0
COG1319	10	C	C	1	0
COG2080	10	C	C	1	0
COG1454	10	C	C	1	0
COG2414	10	C	C	1	0
COG0243	10	C	C	1	0
COG1036	10	C	C	1	0
COG1155	10	C	C	1	0
COG1156	10	C	C	1	0
COG1527	10	C	C	1	0
COG1394	10	C	C	1	0
COG1390	10	C	C	1	0
COG1436	10	C	C	1	0
COG2811	10	C	C	1	0
COG1269	10	C	C	1	0
COG3241	10	C	C	1	0
COG3426	10	C	C	1	0
COG3069	10	C	C	1	0
COG1152	10	C	C	1	0
COG1614	10	C	C	1	0
COG2069	10	C	C	1	0
COG1880	10	C	C	1	0
COG1456	10	C	C	1	0
COG2993	10	C	C	1	0
COG3278	10	C	C	1	0
COG3053	10	C	C	1	0
COG3051	10	C	C	1	0
COG3052	10	C	C	1	0
COG0372	10	C	C	1	0
COG2141	10	C	C	1	0
COG3259	10	C	C	1	0
COG1035	10	C	C	1	0
COG1908	10	C	C	1	0
COG1941	10	C	C	1	0
COG3038	10	C	C	1	0
COG3658	10	C	C	1	0
COG2864	10	C	C	1	0
COG1290	10	C	C	1	0
COG1271	10	C	C	1	0

COG1294	10	C	C	1	0
COG3258	10	C	C	1	0
COG2010	10	C	C	1	0
COG2857	10	C	C	1	0
COG3474	10	C	C	1	0
COG3245	10	C	C	1	0
COG2863	10	C	C	1	0
COG3175	10	C	C	1	0
COG0644	10	C	C	1	0
COG2033	10	C	C	1	0
COG0508	10	C	C	1	0
COG1249	10	C	C	1	0
COG2025	10	C	C	1	0
COG2086	10	C	C	1	0
COG3312	10	C	C	1	0
COG0356	10	C	C	1	0
COG0056	10	C	C	1	0
COG0711	10	C	C	1	0
COG0055	10	C	C	1	0
COG0636	10	C	C	1	0
COG0712	10	C	C	1	0
COG0355	10	C	C	1	0
COG0224	10	C	C	1	0
COG0277	10	C	C	1	0
COG0247	10	C	C	1	0
COG2924	10	CO	C	1	0
COG1625	10	C	C	1	0
COG0731	10	C	C	1	0
COG1032	10	C	C	1	0
COG0437	10	C	C	1	0
COG1142	10	C	C	1	0
COG0633	10	C	C	1	0
COG3411	10	C	C	1	0
COG1141	10	C	C	1	0
COG1145	10	C	C	1	0
COG1146	10	C	C	1	0
COG2440	10	C	C	1	0
COG1018	10	C	C	1	0
COG0716	10	C	C	1	0
COG0651	10	CP	C	1	0
COG0650	10	C	C	1	0
COG1143	10	C	C	1	0
COG1229	10	C	C	1	0
COG1029	10	C	C	1	0
COG2218	10	C	C	1	0

COG1153	10	C	C	1	0
COG2191	10	C	C	1	0
COG2037	10	C	C	1	0
COG0114	10	C	C	1	0
COG3029	10	C	C	1	0
COG3080	10	C	C	1	0
COG1085	10	C	C	1	0
COG0240	10	C	C	1	0
COG0371	10	C	C	1	0
COG0554	10	C	C	1	0
COG0578	10	C	C	1	0
COG0584	10	C	C	1	0
COG2851	10	C	C	1	0
COG1845	10	C	C	1	0
COG3125	10	C	C	1	0
COG0843	10	C	C	1	0
COG1622	10	C	C	1	0
COG1017	10	C	C	1	0
COG1148	10	C	C	1	0
COG2048	10	C	C	1	0
COG1150	10	C	C	1	0
COG0221	10	C	C	1	0
COG3808	10	C	C	1	0
COG1227	10	C	C	1	0
COG0538	10	C	C	1	0
COG2224	10	C	C	1	0
COG1304	10	C	C	1	0
COG1620	10	C	C	1	0
COG1052	10	CHR	C	1	0
COG2225	10	C	C	1	0
COG2055	10	C	C	1	0
COG0039	10	C	C	1	0
COG0281	10	C	C	1	0
COG1149	10	C	C	1	0
COG2838	10	C	C	1	0
COG1927	10	C	C	1	0
COG1251	10	C	C	1	0
COG1012	10	C	C	1	0
COG3288	10	C	C	1	0
COG1282	10	C	C	1	0
COG1252	10	C	C	1	0
COG1034	10	C	C	1	0
COG1902	10	C	C	1	0
COG3761	10	C	C	1	0
COG0377	10	C	C	1	0

COG1905	10	C	C	1	0
COG0852	10	C	C	1	0
COG0649	10	C	C	1	0
COG1005	10	C	C	1	0
COG0713	10	C	C	1	0
COG1007	10	C	C	1	0
COG0838	10	C	C	1	0
COG1008	10	C	C	1	0
COG1009	10	CP	C	1	0
COG0839	10	C	C	1	0
COG1894	10	C	C	1	0
COG0604	10	CR	C	1	0
COG1726	10	C	C	1	0
COG2871	10	C	C	1	0
COG2869	10	C	C	1	0
COG1805	10	C	C	1	0
COG1347	10	C	C	1	0
COG2209	10	C	C	1	0
COG1883	10	C	C	1	0
COG3630	10	C	C	1	0
COG3632	10	C	C	1	0
COG1757	10	C	C	1	0
COG1301	10	C	C	1	0
COG3493	10	C	C	1	0
COG1969	10	C	C	1	0
COG0374	10	C	C	1	0
COG1740	10	C	C	1	0
COG3262	10	C	C	1	0
COG3261	10	C	C	1	0
COG3260	10	C	C	1	0
COG0680	10	C	C	1	0
COG0822	10	C	C	1	0
COG3043	10	C	C	1	0
COG2180	10	C	C	1	0
COG2181	10	C	C	1	0
COG3005	10	C	C	1	0
COG2710	10	C	C	1	0
COG0778	10	C	C	1	0
COG2221	10	C	C	1	0
COG1866	10	C	C	1	0
COG1274	10	C	C	1	0
COG2352	10	C	C	1	0
COG0280	10	C	C	1	0
COG1413	10	C	C	1	0
COG3794	10	C	C	1	0

COG0348	10 C	C	1	0
COG2421	10 C	C	1	0
COG1804	10 C	C	1	0
COG2878	10 C	C	1	0
COG2441	10 C	C	1	0
COG0667	10 C	C	1	0
COG3488	10 C	C	1	0
COG0567	10 C	C	1	0
COG1038	10 C	C	1	0
COG2609	10 C	C	1	0
COG1882	10 C	C	1	0
COG0674	10 C	C	1	0
COG1013	10 C	C	1	0
COG1144	10 C	C	1	0
COG1014	10 C	C	1	0
COG1069	10 C	C	1	0
COG0723	10 C	C	1	0
COG1773	10 C	C	1	0
COG1592	10 C	C	1	0
COG3783	10 C	C	1	0
COG2142	10 C	C	1	0
COG0479	10 C	C	1	0
COG2009	10 C	C	1	0
COG1053	10 C	C	1	0
COG0074	10 C	C	1	0
COG0045	10 C	C	1	0
COG1951	10 C	C	1	0
COG1838	10 C	C	1	0
COG1071	10 C	C	1	0
COG0022	10 C	C	1	0
COG1031	10 C	C	1	0
COG1600	10 C	C	1	0
COG1139	10 C	C	1	0
COG0426	10 C	C	1	0
COG1979	10 C	C	1	0
COG1526	10 C	C	1	0
COG1062	10 C	C	1	0
COG0296	11 G	G	1	0
COG3836	11 G	G	1	0
COG2074	11 G	G	1	0
COG0800	11 G	G	1	0
COG3734	11 G	G	1	0
COG2706	11 G	G	1	0
COG0269	11 G	G	1	0
COG0126	11 G	G	1	0

COG1640	11 G	G	1	0
COG3717	11 G	G	1	0
COG0205	11 G	G	1	0
COG0362	11 G	G	1	0
COG1023	11 G	G	1	0
COG0363	11 G	G	1	0
COG3833	11 G	G	1	0
COG3834	11 G	G	1	0
COG1134	11 G	G	1	0
COG1129	11 G	G	1	0
COG1175	11 G	G	1	0
COG0448	11 G	G	1	0
COG3534	11 G	G	1	0
COG3669	11 G	G	1	0
COG1449	11 G	G	1	0
COG3345	11 G	G	1	0
COG1486	11 G	G	1	0
COG1501	11 G	G	1	0
COG3661	11 G	G	1	0
COG0383	11 G	G	1	0
COG2721	11 G	G	1	0
COG2814	11 G	G	1	0
COG0483	11 G	G	1	0
COG3693	11 G	G	1	0
COG1621	11 G	G	1	0
COG1874	11 G	G	1	0
COG3250	11 G	G	1	0
COG2273	11 G	G	1	0
COG1472	11 G	G	1	0
COG2723	11 G	G	1	0
COG3507	11 G	G	1	0
COG3664	11 G	G	1	0
COG3459	11 G	G	1	0
COG1363	11 G	G	1	0
COG3325	11 G	G	1	0
COG3469	11 G	G	1	0
COG2301	11 G	G	1	0
COG1312	11 G	G	1	0
COG1830	11 G	G	1	0
COG1638	11 G	G	1	0
COG2376	11 G	G	1	0
COG2730	11 G	G	1	0
COG3405	11 G	G	1	0
COG0148	11 G	G	1	0
COG3001	11 G	G	1	0

COG0158	11	G	G	1	0
COG1494	11	G	G	1	0
COG3588	11	G	G	1	0
COG1105	11	G	G	1	0
COG0406	11	G	G	1	0
COG0191	11	G	G	1	0
COG3594	11	G	G	1	0
COG0738	11	G	G	1	0
COG0153	11	G	G	1	0
COG2017	11	G	G	1	0
COG0058	11	G	G	1	0
COG0837	11	G	G	1	0
COG3386	11	G	G	1	0
COG0364	11	G	G	1	0
COG0166	11	G	G	1	0
COG2133	11	G	G	1	0
COG1904	11	G	G	1	0
COG0057	11	G	G	1	0
COG1929	11	G	G	1	0
COG0580	11	G	G	1	0
COG3408	11	G	G	1	0
COG0297	11	G	G	1	0
COG0366	11	G	G	1	0
COG1819	11	GC	G	1	0
COG2610	11	GE	G	1	0
COG3622	11	G	G	1	0
COG2160	11	G	G	1	0
COG2407	11	G	G	1	0
COG3280	11	G	G	1	0
COG2182	11	G	G	1	0
COG0246	11	G	G	1	0
COG0662	11	G	G	1	0
COG1803	11	G	G	1	0
COG3525	11	G	G	1	0
COG1820	11	G	G	1	0
COG2942	11	G	G	1	0
COG2211	11	G	G	1	0
COG1626	11	G	G	1	0
COG2513	11	G	G	1	0
COG0036	11	G	G	1	0
COG1879	11	G	G	1	0
COG0697	11	GER	G	1	0
Permeases	11	GEPRCOG0	G	1	0
COG0574	11	G	G	1	0
COG1080	11	G	G	1	0

COG0033	11 G	G	1	0
COG0588	11 G	G	1	0
COG0696	11 G	G	1	0
COG0279	11 G	G	1	0
COG1109	11 G	G	1	0
COG1482	11 G	G	1	0
COG1015	11 G	G	1	0
COG2190	11 G	G	1	0
COG1264	11 G	G	1	0
COG1263	11 G	G	1	0
COG1447	11 G	G	1	0
COG1440	11 G	G	1	0
COG1455	11 G	G	1	0
COG3414	11 G	G	1	0
COG1445	11 G	G	1	0
COG1762	11 GT	G	1	0
COG3731	11 G	G	1	0
COG3732	11 G	G	1	0
COG3730	11 G	G	1	0
COG1925	11 G	G	1	0
COG1299	11 G	G	1	0
COG3775	11 G	G	1	0
COG2213	11 G	G	1	0
COG2893	11 G	G	1	0
COG3444	11 G	G	1	0
COG3715	11 G	G	1	0
COG3716	11 G	G	1	0
COG2971	11 G	G	1	0
COG2956	11 G	G	1	0
COG2342	11 G	G	1	0
COG2152	11 G	G	1	0
COG3635	11 G	G	1	0
COG0063	11 G	G	1	0
COG0647	11 G	G	1	0
COG0726	11 G	G	1	0
COG1523	11 G	G	1	0
COG3623	11 G	G	1	0
COG3010	11 G	G	1	0
COG3537	11 G	G	1	0
COG2379	11 G	G	1	0
COG0469	11 G	G	1	0
COG0120	11 G	G	1	0
COG0698	11 G	G	1	0
COG1172	11 G	G	1	0
COG1850	11 G	G	1	0

COG0235	11	G	G	1	0
COG1070	11	G	G	1	0
COG1489	11	G	G	1	0
COG0524	11	G	G	1	0
COG0395	11	G	G	1	0
COG1082	11	G	G	1	0
COG2271	11	G	G	1	0
COG1653	11	G	G	1	0
COG3684	11	G	G	1	0
COG2140	11	GR	G	1	0
COG0176	11	G	G	1	0
COG0021	11	G	G	1	0
COG1554	11	G	G	1	0
COG1877	11	G	G	1	0
COG0380	11	G	G	1	0
COG0149	11	G	G	1	0
COG1869	11	G	G	1	0
COG3718	11	G	G	1	0
COG0676	11	G	G	1	0
COG3281	11	G	G	1	0
COG2115	11	G	G	1	0
COG2515	12	E	E	1	0
COG0175	12	EH	E	1	0
COG0722	12	E	E	1	0
COG2876	12	E	E	1	0
COG3200	12	E	E	1	0
COG0710	12	E	E	1	0
COG0757	12	E	E	1	0
COG0337	12	E	E	1	0
COG0065	12	E	E	1	0
COG0066	12	E	E	1	0
COG3185	12	ER	E	1	0
COG0685	12	E	E	1	0
COG3232	12	E	E	1	0
COG0128	12	E	E	1	0
COG0834	12	E	E	1	0
COG0765	12	E	E	1	0
COG0410	12	E	E	1	0
COG0411	12	E	E	1	0
COG0683	12	E	E	1	0
COG0444	12	EP	E	1	0
COG1124	12	EP	E	1	0
COG0747	12	EP	E	1	0
COG0601	12	EP	E	1	0
COG1173	12	EP	E	1	0

COG1126	12	E	E	1	0
COG1125	12	E	E	1	0
COG2113	12	E	E	1	0
COG1174	12	E	E	1	0
COG1176	12	E	E	1	0
COG1177	12	E	E	1	0
COG3842	12	E	E	1	0
COG2061	12	E	E	1	0
COG0040	12	E	E	1	0
COG3705	12	E	E	1	0
COG0440	12	E	E	1	0
COG0548	12	E	E	1	0
COG0002	12	E	E	1	0
COG0624	12	E	E	1	0
COG0686	12	E	E	1	0
COG2049	12	E	E	1	0
COG1984	12	E	E	1	0
COG0814	12	E	E	1	0
COG0531	12	E	E	1	0
COG0833	12	E	E	1	0
COG3579	12	E	E	1	0
COG0308	12	E	E	1	0
COG3075	12	E	E	1	0
COG0547	12	E	E	1	0
COG0147	12	EH	E	1	0
COG0512	12	EH	E	1	0
COG1411	12	E	E	1	0
COG1685	12	EH	E	1	0
COG0010	12	E	E	1	0
COG1166	12	E	E	1	0
COG2235	12	E	E	1	0
COG1438	12	E	E	1	0
COG1982	12	E	E	1	0
COG3138	12	E	E	1	0
COG0165	12	E	E	1	0
COG0137	12	E	E	1	0
COG1446	12	E	E	1	0
COG0367	12	E	E	1	0
COG2502	12	E	E	1	0
COG1027	12	E	E	1	0
COG0136	12	E	E	1	0
COG1448	12	E	E	1	0
COG0527	12	E	E	1	0
COG1362	12	E	E	1	0
COG1775	12	E	E	1	0

COG0559	12 E	E	1	0
COG0115	12 EH	E	1	0
COG1114	12 E	E	1	0
COG0549	12 E	E	1	0
COG0458	12 EF	E	1	0
COG0505	12 EF	E	1	0
COG2939	12 E	E	1	0
COG1605	12 E	E	1	0
COG0082	12 E	E	1	0
COG0626	12 E	E	1	0
COG1104	12 E	E	1	0
COG0031	12 E	E	1	0
COG2362	12 E	E	1	0
COG3048	12 E	E	1	0
COG2195	12 E	E	1	0
COG0019	12 E	E	1	0
COG0253	12 E	E	1	0
COG0289	12 E	E	1	0
COG0329	12 EM	E	1	0
COG0129	12 EG	E	1	0
COG3104	12 E	E	1	0
COG1506	12 E	E	1	0
COG3192	12 E	E	1	0
COG1037	12 E	E	1	0
COG1113	12 E	E	1	0
COG0014	12 E	E	1	0
COG0405	12 E	E	1	0
COG0263	12 E	E	1	0
COG0076	12 E	E	1	0
COG0334	12 E	E	1	0
COG3643	12 E	E	1	0
COG0067	12 E	E	1	0
COG0069	12 E	E	1	0
COG0070	12 E	E	1	0
COG2066	12 E	E	1	0
COG0118	12 E	E	1	0
COG0174	12 E	E	1	0
COG0754	12 E	E	1	0
COG0509	12 E	E	1	0
COG0404	12 E	E	1	0
COG1003	12 E	E	1	0
COG0403	12 E	E	1	0
COG2716	12 E	E	1	0
COG0112	12 E	E	1	0
COG0665	12 E	E	1	0

COG2986	12	E	E	1	0
COG0141	12	E	E	1	0
COG1387	12	ER	E	1	0
COG0241	12	E	E	1	0
COG0079	12	E	E	1	0
COG2040	12	E	E	1	0
COG2021	12	E	E	1	0
COG0460	12	E	E	1	0
COG0083	12	E	E	1	0
COG1897	12	E	E	1	0
COG0131	12	E	E	1	0
COG0107	12	E	E	1	0
COG0134	12	E	E	1	0
COG0473	12	E	E	1	0
COG0119	12	E	E	1	0
COG0059	12	EH	E	1	0
COG3844	12	E	E	1	0
COG3191	12	EQ	E	1	0
COG0252	12	EJ	E	1	0
COG1760	12	E	E	1	0
COG0346	12	E	E	1	0
COG0260	12	E	E	1	0
COG2309	12	E	E	1	0
COG1509	12	E	E	1	0
COG2755	12	E	E	1	0
COG3404	12	E	E	1	0
COG0646	12	E	E	1	0
COG1410	12	E	E	1	0
COG0620	12	E	E	1	0
COG3799	12	E	E	1	0
COG1231	12	E	E	1	0
COG1834	12	E	E	1	0
COG1246	12	E	E	1	0
COG3741	12	E	E	1	0
COG0145	12	E	E	1	0
COG0146	12	E	E	1	0
COG2902	12	E	E	1	0
COG0493	12	ER	E	1	0
COG1115	12	E	E	1	0
COG0786	12	E	E	1	0
COG0591	12	EHR	E	1	0
COG3633	12	E	E	1	0
COG0347	12	E	E	1	0
COG2873	12	E	E	1	0
COG1164	12	E	E	1	0

COG0078	12 E	E	1	0
COG1168	12 E	E	1	0
COG0160	12 E	E	1	0
COG0436	12 E	E	1	0
COG3340	12 E	E	1	0
COG2957	12 E	E	1	0
COG3186	12 E	E	1	0
COG0111	12 E	E	1	0
COG0139	12 E	E	1	0
COG0140	12 E	E	1	0
COG0135	12 E	E	1	0
COG0106	12 E	E	1	0
COG0560	12 E	E	1	0
COG2856	12 E	E	1	0
COG1465	12 E	E	1	0
COG3616	12 E	E	1	0
COG3457	12 E	E	1	0
COG1296	12 E	E	1	0
COG1687	12 E	E	1	0
COG2866	12 E	E	1	0
COG2423	12 E	E	1	0
COG0077	12 E	E	1	0
COG0287	12 E	E	1	0
COG0506	12 E	E	1	0
COG1770	12 E	E	1	0
COG1703	12 E	E	1	0
COG1280	12 E	E	1	0
COG0345	12 E	E	1	0
COG1586	12 E	E	1	0
COG1748	12 E	E	1	0
COG0520	12 E	E	1	0
COG1921	12 E	E	1	0
COG0709	12 E	E	1	0
COG1045	12 E	E	1	0
COG1505	12 E	E	1	0
COG0075	12 E	E	1	0
COG0169	12 E	E	1	0
COG0703	12 E	E	1	0
COG0421	12 E	E	1	0
COG0687	12 E	E	1	0
COG3724	12 E	E	1	0
COG2988	12 E	E	1	0
COG2171	12 E	E	1	0
COG0028	12 EH	E	1	0
COG2008	12 E	E	1	0

COG1171	12	E	E	1	0
COG1063	12	ER	E	1	0
COG0498	12	E	E	1	0
COG3060	12	EK	E	1	0
COG1305	12	E	E	1	0
COG3483	12	E	E	1	0
COG0159	12	E	E	1	0
COG0133	12	E	E	1	0
COG3033	12	E	E	1	0
COG2981	12	E	E	1	0
COG0804	12	E	E	1	0
COG0832	12	E	E	1	0
COG0831	12	E	E	1	0
COG3194	12	E	E	1	0
COG2987	12	E	E	1	0
COG3591	12	E	E	1	0
Various	12	EPGRCOG1	E	1	0
COG0006	12	E	E	1	0
COG3227	12	E	E	1	0
COG2317	12	E	E	1	0
COG2355	12	E	E	1	0
COG0339	12	E	E	1	0
COG0737	13	F	F	1	0
COG1051	13	F	F	1	0
COG0138	13	F	F	1	0
COG1001	13	F	F	1	0
COG0503	13	F	F	1	0
COG1816	13	F	F	1	0
COG2169	13	F	F	1	0
COG3072	13	F	F	1	0
COG1437	13	F	F	1	0
COG0563	13	F	F	1	0
COG0015	13	F	F	1	0
COG0104	13	F	F	1	0
COG2019	13	F	F	1	0
COG1781	13	F	F	1	0
COG0540	13	F	F	1	0
COG0504	13	F	F	1	0
COG0295	13	F	F	1	0
COG0283	13	F	F	1	0
COG1102	13	F	F	1	0
COG0402	13	FR	F	1	0
COG0590	13	FJ	F	1	0
COG1953	13	FH	F	1	0
COG1864	13	F	F	1	0

COG0717	13	F	F	1	0
COG2131	13	F	F	1	0
COG1428	13	F	F	1	0
COG0274	13	F	F	1	0
COG0537	13	FGR	F	1	0
COG0418	13	F	F	1	0
COG0044	13	F	F	1	0
COG0167	13	F	F	1	0
COG0248	13	FP	F	1	0
COG0299	13	F	F	1	0
COG0027	13	F	F	1	0
COG0788	13	F	F	1	0
COG2759	13	F	F	1	0
COG0518	13	F	F	1	0
COG0519	13	F	F	1	0
COG0034	13	F	F	1	0
COG0194	13	F	F	1	0
COG0634	13	F	F	1	0
COG0516	13	F	F	1	0
COG1957	13	F	F	1	0
COG3613	13	F	F	1	0
COG0105	13	F	F	1	0
COG1972	13	F	F	1	0
COG0775	13	F	F	1	0
COG1949	13	F	F	1	0
COG0461	13	F	F	1	0
COG0856	13	F	F	1	0
COG0284	13	F	F	1	0
COG1328	13	F	F	1	0
COG0151	13	F	F	1	0
COG0150	13	F	F	1	0
COG0026	13	F	F	1	0
COG0152	13	F	F	1	0
COG0041	13	F	F	1	0
COG1828	13	F	F	1	0
COG0047	13	F	F	1	0
COG0046	13	F	F	1	0
COG0462	13	FE	F	1	0
COG1351	13	F	F	1	0
COG1936	13	F	F	1	0
COG1780	13	F	F	1	0
COG0005	13	F	F	1	0
COG1457	13	F	F	1	0
COG0813	13	F	F	1	0
COG2065	13	F	F	1	0

COG0209	13 F	F	1	0
COG0208	13 F	F	1	0
COG1435	13 F	F	1	0
COG0213	13 F	F	1	0
COG0125	13 F	F	1	0
COG0207	13 F	F	1	0
COG2022	13 F	F	1	0
COG0035	13 F	F	1	0
COG0572	13 F	F	1	0
COG2820	13 F	F	1	0
COG0528	13 F	F	1	0
COG2233	13 F	F	1	0
COG0127	13 F	F	1	0
COG0232	13 F	F	1	0
COG0756	13 F	F	1	0
COG1575	14 H	H	1	0
COG2227	14 H	H	1	0
COG0654	14 HC	H	1	0
COG0543	14 HC	H	1	0
COG1165	14 H	H	1	0
COG0108	14 H	H	1	0
COG0163	14 H	H	1	0
COG0043	14 H	H	1	0
COG3161	14 H	H	1	0
COG0382	14 H	H	1	0
COG0190	14 H	H	1	0
COG0212	14 H	H	1	0
COG0720	14 H	H	1	0
COG0801	14 H	H	1	0
COG0156	14 H	H	1	0
COG1840	14 H	H	1	0
COG1178	14 H	H	1	0
COG3840	14 H	H	1	0
COG2109	14 H	H	1	0
COG2087	14 H	H	1	0
COG0161	14 H	H	1	0
COG1731	14 H	H	1	0
COG0853	14 H	H	1	0
COG0029	14 H	H	1	0
COG0502	14 H	H	1	0
COG0340	14 H	H	1	0
COG1903	14 H	H	1	0
COG2073	14 H	H	1	0
COG0310	14 H	H	1	0
COG1270	14 H	H	1	0

COG1429	14 H	H	1	0
COG0368	14 H	H	1	0
COG1492	14 H	H	1	0
COG1797	14 H	H	1	0
COG1541	14 H	H	1	0
COG0408	14 H	H	1	0
COG0635	14 H	H	1	0
COG0113	14 H	H	1	0
COG0684	14 H	H	1	0
COG1154	14 HI	H	1	0
COG0237	14 H	H	1	0
COG0132	14 H	H	1	0
COG0262	14 H	H	1	0
COG1539	14 H	H	1	0
COG0294	14 H	H	1	0
COG0447	14 H	H	1	0
COG1179	14 H	H	1	0
COG0476	14 H	H	1	0
COG0196	14 H	H	1	0
COG0285	14 H	H	1	0
COG0302	14 H	H	1	0
COG0807	14 H	H	1	0
COG2266	14 H	H	1	0
COG2918	14 H	H	1	0
COG3572	14 H	H	1	0
COG0142	14 H	H	1	0
COG0001	14 H	H	1	0
COG0373	14 H	H	1	0
COG0189	14 HJ	H	1	0
COG2145	14 H	H	1	0
COG0351	14 H	H	1	0
COG1169	14 HQ	H	1	0
COG0413	14 H	H	1	0
COG1893	14 H	H	1	0
COG0320	14 H	H	1	0
COG0095	14 H	H	1	0
COG0321	14 H	H	1	0
COG1477	14 H	H	1	0
COG3252	14 H	H	1	0
COG2226	14 H	H	1	0
COG1240	14 H	H	1	0
COG1239	14 H	H	1	0
COG0315	14 H	H	1	0
COG2896	14 H	H	1	0
COG0303	14 H	H	1	0

COG0521	14 H	H	1	0
COG0314	14 H	H	1	0
COG1977	14 H	H	1	0
COG3585	14 H	H	1	0
COG1763	14 H	H	1	0
COG0746	14 H	H	1	0
COG1962	14 H	H	1	0
COG0171	14 H	H	1	0
COG0846	14 H	H	1	0
COG2038	14 H	H	1	0
COG1056	14 H	H	1	0
COG3201	14 H	H	1	0
COG0157	14 H	H	1	0
COG1057	14 H	H	1	0
COG1488	14 H	H	1	0
COG1441	14 H	H	1	0
COG1072	14 H	H	1	0
COG0414	14 H	H	1	0
COG0669	14 H	H	1	0
COG0452	14 H	H	1	0
COG3697	14 HI	H	1	0
COG1932	14 HE	H	1	0
COG2045	14 HR	H	1	0
COG1424	14 H	H	1	0
COG0109	14 H	H	1	0
COG0181	14 H	H	1	0
COG2082	14 H	H	1	0
COG2243	14 H	H	1	0
COG1010	14 H	H	1	0
COG2875	14 H	H	1	0
COG2241	14 H	H	1	0
COG2242	14 H	H	1	0
COG2099	14 H	H	1	0
COG3172	14 H	H	1	0
COG1713	14 H	H	1	0
COG0311	14 H	H	1	0
COG0276	14 H	H	1	0
COG1232	14 H	H	1	0
COG2154	14 H	H	1	0
COG2978	14 H	H	1	0
COG0854	14 H	H	1	0
COG1995	14 H	H	1	0
COG2240	14 H	H	1	0
COG0259	14 H	H	1	0
COG0214	14 H	H	1	0

COG0117	14 H	H	1	0
COG1985	14 H	H	1	0
COG0379	14 H	H	1	0
COG0307	14 H	H	1	0
COG0054	14 H	H	1	0
COG0499	14 H	H	1	0
COG0192	14 H	H	1	0
COG1648	14 H	H	1	0
COG2104	14 H	H	1	0
COG0301	14 H	H	1	0
COG1060	14 HR	H	1	0
COG0422	14 H	H	1	0
COG0611	14 H	H	1	0
COG0352	14 H	H	1	0
COG1564	14 H	H	1	0
COG1767	14 H	H	1	0
COG2941	14 H	H	1	0
COG3071	14 H	H	1	0
COG0407	14 H	H	1	0
COG0007	14 H	H	1	0
COG1587	14 H	H	1	0
COG0331	15 I	I	1	0
COG0204	15 I	I	1	0
COG0743	15 I	I	1	0
COG0245	15 I	I	1	0
COG1250	15 I	I	1	0
COG3425	15 I	I	1	0
COG2084	15 I	I	1	0
COG0764	15 I	I	1	0
COG0304	15 IQ	I	1	0
COG0332	15 I	I	1	0
COG1211	15 I	I	1	0
COG1947	15 I	I	1	0
COG1133	15 I	I	1	0
COG0183	15 I	I	1	0
COG0825	15 I	I	1	0
COG0777	15 I	I	1	0
COG1924	15 I	I	1	0
COG1788	15 I	I	1	0
COG2057	15 I	I	1	0
COG0236	15 IQ	I	1	0
COG1182	15 I	I	1	0
COG1960	15 I	I	1	0
COG1607	15 I	I	1	0
COG0318	15 IQ	I	1	0

COG1946	15 I	I	1	0
COG0365	15 I	I	1	0
COG0511	15 I	I	1	0
COG0439	15 I	I	1	0
COG2134	15 I	I	1	0
COG0575	15 I	I	1	0
COG2272	15 I	I	1	0
COG0170	15 I	I	1	0
COG1024	15 I	I	1	0
COG0623	15 I	I	1	0
COG0657	15 I	I	1	0
COG3239	15 I	I	1	0
COG0416	15 I	I	1	0
COG1398	15 I	I	1	0
COG2937	15 I	I	1	0
COG1257	15 I	I	1	0
COG1443	15 I	I	1	0
COG1022	15 I	I	1	0
COG2067	15 I	I	1	0
COG2267	15 I	I	1	0
COG0671	15 I	I	1	0
COG2185	15 I	I	1	0
COG1884	15 I	I	1	0
COG1577	15 I	I	1	0
COG3407	15 I	I	1	0
COG1260	15 I	I	1	0
COG2867	15 I	I	1	0
COG0761	15 IM	I	1	0
COG1842	15 I	I	1	0
COG1267	15 I	I	1	0
COG0558	15 I	I	1	0
COG0688	15 I	I	1	0
COG1183	15 I	I	1	0
COG1502	15 I	I	1	0
COG3240	15 I	I	1	0
COG0736	15 I	I	1	0
COG1562	15 I	I	1	0
COG3243	15 I	I	1	0
COG1870	15 I	I	1	0
COG2030	15 I	I	1	0
COG1835	15 I	I	1	0
COG3675	15 I	I	1	0
COG3154	15 I	I	1	0
COG3255	15 I	I	1	0
COG2031	15 I	I	1	0

COG1657	15 I	I	1	0
COG3000	15 I	I	1	0
COG0020	15 I	I	1	0
COG0179	16 Q	Q	1	0
COG2274	16 Q	Q	1	0
COG1131	16 Q	Q	1	0
COG1132	16 Q	Q	1	0
COG3458	16 Q	Q	1	0
COG2015	16 Q	Q	1	0
COG3527	16 Q	Q	1	0
COG1335	16 Q	Q	1	0
COG2746	16 Q	Q	1	0
COG2368	16 Q	Q	1	0
COG3433	16 Q	Q	1	0
COG2162	16 Q	Q	1	0
COG0841	16 Q	Q	1	0
COG3510	16 Q	Q	1	0
COG3733	16 Q	Q	1	0
COG2124	16 Q	Q	1	0
COG1028	16 QR	Q	1	0
COG0412	16 Q	Q	1	0
COG3435	16 Q	Q	1	0
COG3508	16 Q	Q	1	0
COG1228	16 Q	Q	1	0
COG1535	16 Q	Q	1	0
COG3670	16 Q	Q	1	0
COG3486	16 Q	Q	1	0
COG3473	16 Q	Q	1	0
COG0845	16 Q	Q	1	0
COG1566	16 Q	Q	1	0
COG3653	16 Q	Q	1	0
COG0534	16 Q	Q	1	0
COG1020	16 Q	Q	1	0
COG3315	16 Q	Q	1	0
COG1021	16 Q	Q	1	0
COG3479	16 Q	Q	1	0
COG2091	16 Q	Q	1	0
COG2977	16 Q	Q	1	0
COG1233	16 Q	Q	1	0
COG3509	16 Q	Q	1	0
COG3321	16 Q	Q	1	0
COG2761	16 Q	Q	1	0
COG3424	16 Q	Q	1	0
COG3208	16 Q	Q	1	0
COG2175	16 Q	Q	1	0

COG3485	16 Q	Q	1	0
COG3320	16 Q	Q	1	0
COG2132	16 Q	Q	1	0
COG3207	16 Q	Q	1	0
COG2931	16 Q	Q	1	0
COG0500	16 QR	Q	1	0
COG3570	16 Q	Q	1	0
COG3319	16 Q	Q	1	0
COG3284	16 Q	Q	1	0
COG2313	16 Q	Q	1	0
COG3460	16 Q	Q	1	0
COG3135	16 Q	Q	1	0
COG2050	16 Q	Q	1	0
COG2251	17 R	R	1	0
COG1137	17 R	R	1	0
COG0842	17 R	R	1	0
COG0767	17 R	R	1	0
COG1277	17 R	R	1	0
COG1136	17 R	R	1	0
COG0577	17 R	R	1	0
COG3845	17 R	R	1	0
COG1464	17 R	R	1	0
COG1707	17 R	R	1	0
COG2344	17 R	R	1	0
COG1759	17 R	R	1	0
COG1606	17 R	R	1	0
COG0488	17 R	R	1	0
COG1123	17 R	R	1	0
COG1855	17 R	R	1	0
COG0456	17 R	R	1	0
COG0110	17 R	R	1	0
COG3700	17 R	R	1	0
COG3445	17 R	R	1	0
COG0656	17 R	R	1	0
COG2704	17 R	R	1	0
COG0666	17 R	R	1	0
COG1103	17 R	R	1	0
COG1938	17 R	R	1	0
COG1750	17 R	R	1	0
COG0641	17 R	R	1	0
COG1512	17 R	R	1	0
COG0857	17 R	R	1	0
COG2516	17 R	R	1	0
COG1779	17 R	R	1	0
COG0517	17 R	R	1	0

COG0663	17 R	R	1	0
COG1853	17 R	R	1	0
COG3302	17 R	R	1	0
COG2118	17 R	R	1	0
COG2916	17 R	R	1	0
COG3046	17 R	R	1	0
COG2070	17 R	R	1	0
COG1205	17 R	R	1	0
COG0073	17 R	R	1	0
COG0780	17 R	R	1	0
COG2312	17 R	R	1	0
COG1647	17 R	R	1	0
COG0618	17 R	R	1	0
COG3401	17 R	R	1	0
COG1635	17 R	R	1	0
COG1100	17 R	R	1	0
COG1159	17 R	R	1	0
COG2262	17 R	R	1	0
COG3729	17 R	R	1	0
COG1078	17 R	R	1	0
COG2346	17 R	R	1	0
COG3157	17 R	R	1	0
COG1331	17 R	R	1	0
COG1073	17 R	R	1	0
COG0670	17 R	R	1	0
COG0396	17 R	R	1	0
COG3491	17 R	R	1	0
COG3631	17 R	R	1	0
COG2373	17 R	R	1	0
COG1201	17 R	R	1	0
COG1279	17 R	R	1	0
COG2244	17 R	R	1	0
COG1473	17 R	R	1	0
COG3568	17 R	R	1	0
COG1235	17 R	R	1	0
COG1237	17 R	R	1	0
COG1234	17 R	R	1	0
COG0714	17 R	R	1	0
COG0655	17 R	R	1	0
COG2005	17 R	R	1	0
COG1691	17 R	R	1	0
COG0733	17 R	R	1	0
COG3081	17 R	R	1	0
COG3291	17 R	R	1	0
COG3008	17 R	R	1	0

COG3621	17	R	R	1	0
COG3331	17	R	R	1	0
COG1463	17	R	R	1	0
COG2011	17	R	R	1	0
COG2252	17	R	R	1	0
COG2270	17	R	R	1	0
COG3628	17	R	R	1	0
COG3740	17	R	R	1	0
COG3500	17	R	R	1	0
COG3499	17	R	R	1	0
COG3497	17	R	R	1	0
COG3498	17	R	R	1	0
COG1783	17	R	R	1	0
COG3772	17	R	R	1	0
COG1881	17	R	R	1	0
COG1741	17	R	R	1	0
COG2446	17	R	R	1	0
COG2232	17	R	R	1	0
COG1821	17	R	R	1	0
COG0433	17	R	R	1	0
COG1485	17	R	R	1	0
COG2603	17	R	R	1	0
COG3106	17	R	R	1	0
COG3378	17	R	R	1	0
COG0603	17	R	R	1	0
COG1672	17	R	R	1	0
COG1483	17	R	R	1	0
COG2607	17	R	R	1	0
COG3044	17	R	R	1	0
COG0802	17	R	R	1	0
COG1106	17	R	R	1	0
COG2102	17	R	R	1	0
COG1618	17	R	R	1	0
COG1832	17	R	R	1	0
COG3413	17	R	R	1	0
COG3849	17	R	R	1	0
COG1571	17	R	R	1	0
COG1342	17	R	R	1	0
COG1661	17	R	R	1	0
COG2100	17	R	R	1	0
COG0535	17	R	R	1	0
COG1244	17	R	R	1	0
COG1964	17	R	R	1	0
COG3313	17	R	R	1	0
COG2000	17	R	R	1	0

COG0727	17 R	R	1	0
COG0820	17 R	R	1	0
COG0012	17 R	R	1	0
COG0486	17 R	R	1	0
COG0536	17 R	R	1	0
COG1084	17 R	R	1	0
COG1163	17 R	R	1	0
COG2229	17 R	R	1	0
COG3596	17 R	R	1	0
COG1341	17 R	R	1	0
COG0218	17 R	R	1	0
COG1160	17 R	R	1	0
COG1161	17 R	R	1	0
COG1162	17 R	R	1	0
COG0699	17 R	R	1	0
COG1418	17 R	R	1	0
COG2316	17 R	R	1	0
COG3810	17 R	R	1	0
COG3481	17 R	R	1	0
COG1407	17 R	R	1	0
COG2907	17 R	R	1	0
COG3380	17 R	R	1	0
COG0385	17 R	R	1	0
COG1823	17 R	R	1	0
COG1444	17 R	R	1	0
COG1660	17 R	R	1	0
COG2183	17 R	R	1	0
COG1847	17 R	R	1	0
COG1837	17 R	R	1	0
COG1094	17 R	R	1	0
COG3688	17 R	R	1	0
COG1818	17 R	R	1	0
COG1532	17 R	R	1	0
COG1537	17 R	R	1	0
COG1611	17 R	R	1	0
COG0220	17 R	R	1	0
COG2384	17 R	R	1	0
COG2933	17 R	R	1	0
COG1092	17 R	R	1	0
COG3129	17 R	R	1	0
COG0434	17 R	R	1	0
COG0042	17 R	R	1	0
COG2220	17 R	R	1	0
COG0612	17 R	R	1	0
COG1026	17 R	R	1	0

COG2738	17 R	R	1	0
COG1913	17 R	R	1	0
COG0312	17 R	R	1	0
COG2388	17 R	R	1	0
COG3153	17 R	R	1	0
COG3393	17 R	R	1	0
COG3818	17 R	R	1	0
COG1075	17 R	R	1	0
COG2936	17 R	R	1	0
COG2153	17 R	R	1	0
COG1350	17 R	R	1	0
COG0388	17 R	R	1	0
COG1040	17 R	R	1	0
COG3173	17 R	R	1	0
COG0354	17 R	R	1	0
COG2234	17 R	R	1	0
COG1608	17 R	R	1	0
COG1829	17 R	R	1	0
COG2521	17 R	R	1	0
COG1907	17 R	R	1	0
COG3577	17 R	R	1	0
COG3179	17 R	R	1	0
COG3233	17 R	R	1	0
COG3608	17 R	R	1	0
COG0579	17 R	R	1	0
COG0673	17 R	R	1	0
COG2085	17 R	R	1	0
COG1712	17 R	R	1	0
COG1355	17 R	R	1	0
COG3565	17 R	R	1	0
COG3271	17 R	R	1	0
COG3341	17 R	R	1	0
COG2409	17 R	R	1	0
COG3450	17 R	R	1	0
COG3324	17 R	R	1	0
COG0325	17 R	R	1	0
COG0384	17 R	R	1	0
COG0400	17 R	R	1	0
COG0627	17 R	R	1	0
COG3150	17 R	R	1	0
COG1752	17 R	R	1	0
COG3545	17 R	R	1	0
COG1033	17 R	R	1	0
COG2374	17 R	R	1	0
COG3467	17 R	R	1	0

COG3576	17 R	R	1	0
COG0431	17 R	R	1	0
COG2081	17 R	R	1	0
COG0121	17 R	R	1	0
COG3442	17 R	R	1	0
COG2071	17 R	R	1	0
COG1216	17 R	R	1	0
COG1203	17 R	R	1	0
COG1204	17 R	R	1	0
COG1719	17 R	R	1	0
COG3083	17 R	R	1	0
COG2179	17 R	R	1	0
COG1353	17 R	R	1	0
COG2819	17 R	R	1	0
COG2945	17 R	R	1	0
COG0429	17 R	R	1	0
COG3571	17 R	R	1	0
COG0595	17 R	R	1	0
COG2248	17 R	R	1	0
COG1896	17 R	R	1	0
COG2333	17 R	R	1	0
COG0561	17 R	R	1	0
COG1011	17 R	R	1	0
COG3769	17 R	R	1	0
COG0596	17 R	R	1	0
COG0392	17 R	R	1	0
COG0061	17 R	R	1	0
COG0645	17 R	R	1	0
COG1597	17 R	R	1	0
COG1461	17 R	R	1	0
COG2605	17 R	R	1	0
COG1364	17 R	R	1	0
COG3607	17 R	R	1	0
COG0719	17 R	R	1	0
COG1584	17 R	R	1	0
COG3641	17 R	R	1	0
COG1272	17 R	R	1	0
COG1480	17 R	R	1	0
COG2194	17 R	R	1	0
COG1988	17 R	R	1	0
COG3318	17 R	R	1	0
COG1399	17 R	R	1	0
COG1782	17 R	R	1	0
COG0319	17 R	R	1	0
COG1451	17 R	R	1	0

COG3687	17 R	R	1	0
COG2159	17 R	R	1	0
COG3618	17 R	R	1	0
COG1574	17 R	R	1	0
COG1735	17 R	R	1	0
COG1831	17 R	R	1	0
COG2872	17 R	R	1	0
COG1099	17 R	R	1	0
COG1266	17 R	R	1	0
COG0613	17 R	R	1	0
COG2151	17 R	R	1	0
COG2321	17 R	R	1	0
COG2520	17 R	R	1	0
COG0313	17 R	R	1	0
COG1568	17 R	R	1	0
COG1295	17 R	R	1	0
COG0658	17 R	R	1	0
COG3582	17 R	R	1	0
COG1439	17 R	R	1	0
COG1367	17 R	R	1	0
COG1487	17 R	R	1	0
COG1569	17 R	R	1	0
COG1848	17 R	R	1	0
COG2402	17 R	R	1	0
COG2405	17 R	R	1	0
COG1623	17 R	R	1	0
COG1545	17 R	R	1	0
COG3478	17 R	R	1	0
COG3529	17 R	R	1	0
COG1090	17 R	R	1	0
COG1548	17 R	R	1	0
COG1058	17 R	R	1	0
COG1019	17 R	R	1	0
COG2413	17 R	R	1	0
COG3541	17 R	R	1	0
COG1669	17 R	R	1	0
COG1708	17 R	R	1	0
COG3573	17 R	R	1	0
COG3560	17 R	R	1	0
COG1453	17 R	R	1	0
COG2358	17 R	R	1	0
COG2998	17 R	R	1	0
COG2823	17 R	R	1	0
COG3443	17 R	R	1	0
COG1427	17 R	R	1	0

COG1559	17	R	R	1	0
COG2107	17	R	R	1	0
COG0390	17	R	R	1	0
COG0628	17	R	R	1	0
COG2056	17	R	R	1	0
COG2985	17	R	R	1	0
COG3329	17	R	R	1	0
COG3368	17	R	R	1	0
COG0679	17	R	R	1	0
COG0701	17	R	R	1	0
COG0730	17	R	R	1	0
COG0795	17	R	R	1	0
COG2962	17	R	R	1	0
COG2044	17	R	R	1	0
COG3211	17	R	R	1	0
COG0637	17	R	R	1	0
COG0546	17	R	R	1	0
COG1646	17	R	R	1	0
COG0622	17	R	R	1	0
COG2129	17	R	R	1	0
COG2404	17	R	R	1	0
COG1408	17	R	R	1	0
COG1409	17	R	R	1	0
COG1768	17	R	R	1	0
COG1926	17	R	R	1	0
COG2236	17		R	0	1
COG3178	17	R	R	1	0
COG1694	17	R	R	1	0
COG2150	17	R	R	1	0
COG2514	17	R	R	1	0
COG1827	17	R	R	1	0
COG2103	17	R	R	1	0
COG0824	17	R	R	1	0
COG2522	17	R	R	1	0
COG3054	17	R	R	1	0
COG3800	17	R	R	1	0
COG1800	17	R	R	1	0
COG2391	17	R	R	1	0
COG0661	17	R	R	1	0
COG3030	17	R	R	1	0
COG2304	17	R	R	1	0
COG2961	17	R	R	1	0
COG2366	17	R	R	1	0
COG2940	17	R	R	1	0
COG0523	17	R	R	1	0

COG2910	17 R	R	1	0
COG2130	17 R	R	1	0
COG2249	17 R	R	1	0
COG3180	17 R	R	1	0
COG1380	17 R	R	1	0
COG2334	17 R	R	1	0
COG0693	17 R	R	1	0
COG3107	17 R	R	1	0
COG1245	17 R	R	1	0
COG3113	17 R	R	1	0
COG2144	17 R	R	1	0
COG0300	17 R	R	1	0
COG1202	17 R	R	1	0
COG2969	17 R	R	1	0
COG2166	17 R	R	1	0
COG0496	17 R	R	1	0
COG0790	17 R	R	1	0
COG0457	17 R	R	1	0
COG2351	17 R	R	1	0
COG1135	17 R	R	1	0
COG1079	17 R	R	1	0
COG2401	17 R	R	1	0
COG1268	17 R	R	1	0
COG2108	17 R	R	1	0
COG1923	17 R	R	1	0
COG1878	17 R	R	1	0
COG2018	17 R	R	1	0
COG2842	17 R	R	1	0
COG1373	17 R	R	1	0
COG1365	17 R	R	1	0
COG2517	17 R	R	1	0
COG2137	17 R	R	1	0
COG3788	17 R	R	1	0
COG1754	17 R	R	1	0
COG2509	17 R	R	1	0
COG2768	17 R	R	1	0
COG1242	17 R	R	1	0
COG3217	17 R	R	1	0
COG1313	17 R	R	1	0
COG2992	17 R	R	1	0
COG2068	17 R	R	1	0
COG0446	17 R	R	1	0
COG1634	17 R	R	1	0
COG1645	17 R	R	1	0
COG3383	17 R	R	1	0

COG2054	17	R	R	1	0
COG3381	17	R	R	1	0
COG2329	17	R	R	1	0
COG1054	17	R	R	1	0
COG2041	17	R	R	1	0
COG2311	17	R	R	1	0
COG2364	17	R	R	1	0
COG0705	17	R	R	1	0
COG3136	17	R	R	1	0
COG1811	17	R	R	1	0
COG0728	17	R	R	1	0
COG1287	17	R	R	1	0
COG1286	17	R	R	1	0
COG2715	17	R	R	1	0
COG1546	17	R	R	1	0
COG1999	17	R	R	1	0
COG2079	17	R	R	1	0
COG2915	17	R	R	1	0
COG3550	17	R	R	1	0
COG1942	17	R	R	1	0
COG2372	17	R	R	1	0
COG1402	17	R	R	1	0
COG2802	17	R	R	1	0
COG1778	17	R	R	1	0
COG1412	17	R	R	1	0
COG1524	17	R	R	1	0
COG2047	17	R	R	1	0
COG1540	17	R	R	1	0
COG3552	17	R	R	1	0
COG2319	17	R	R	1	0
COG0375	17	R	R	1	0
COG1064	17	R	R	1	0
COG0491	17	R	R	1	0
COG1994	17	R	R	1	0
COG2158	17	R	R	1	0
COG3364	17	R	R	1	0
COG1579	17	R	R	1	0
COG1593	17	R	R	1	0
COG2095	17	S	R	0	1
COG1822	18	S	S	1	0
COG0762	18	S	S	1	0
COG3548	18	S	S	1	0
COG0344	18	S	S	1	0
COG1470	18	S	S	1	0
COG1511	18	S	S	1	0

COG1557	18	S	S	1	0
COG1784	18	S	S	1	0
COG1808	18	S	S	1	0
COG1836	18	S	S	1	0
COG1950	18	S	S	1	0
COG1971	18	S	S	1	0
COG1981	18	S	S	1	0
COG2035	18	S	S	1	0
COG2064	18	S	S	1	0
COG2119	18	S	S	1	0
COG2149	18	S	S	1	0
COG2215	18	S	S	1	0
COG2237	18	S	S	1	0
COG2259	18	S	S	1	0
COG2314	18	S	S	1	0
COG2322	18	S	S	1	0
COG2323	18	S	S	1	0
COG2324	18	S	S	1	0
COG2510	18	S	S	1	0
COG2860	18	S	S	1	0
COG3326	18	S	S	1	0
COG3336	18	S	S	1	0
COG3356	18	S	S	1	0
COG3371	18	S	S	1	0
COG3374	18	S	S	1	0
COG3428	18	S	S	1	0
COG3431	18	S	S	1	0
COG3462	18	S	S	1	0
COG3503	18	S	S	1	0
COG3556	18	S	S	1	0
COG3647	18	S	S	1	0
COG3650	18	S	S	1	0
COG3671	18	S	S	1	0
COG3689	18	S	S	1	0
COG3694	18	S	S	1	0
COG3759	18	S	S	1	0
COG3762	18	S	S	1	0
COG3766	18	S	S	1	0
COG3768	18	S	S	1	0
COG3771	18	S	S	1	0
COG3776	18	S	S	1	0
COG3781	18	S	S	1	0
COG3815	18	S	S	1	0
COG3817	18	S	S	1	0
COG3819	18	S	S	1	0

COG1967	18	S	S	1	0
COG2261	18	S	S	1	0
COG3019	18	S	S	1	0
COG3652	18	S	S	1	0
COG3683	18	S	S	1	0
COG3656	18	S	S	1	0
COG3672	18	S	S	1	0
COG3698	18	S	S	1	0
COG3471	18	S	S	1	0
COG3477	18	S	S	1	0
COG3466	18	S	S	1	0
COG3702	18	S	S	1	0
COG0011	18	S	S	1	0
COG0062	18	S	S	1	0
COG0217	18	S	S	1	0
COG0316	18	S	S	1	0
COG0327	18	S	S	1	0
COG0391	18	S	S	1	0
COG0393	18	S	S	1	0
COG0397	18	S	S	1	0
COG0398	18	S	S	1	0
COG0432	18	S	S	1	0
COG0585	18	S	S	1	0
COG0759	18	S	S	1	0
COG1259	18	S	S	1	0
COG1273	18	S	S	1	0
COG1306	18	S	S	1	0
COG1323	18	S	S	1	0
COG1332	18	S	S	1	0
COG1336	18	S	S	1	0
COG1337	18	S	S	1	0
COG1343	18	S	S	1	0
COG1354	18	S	S	1	0
COG1359	18	S	S	1	0
COG1371	18	S	S	1	0
COG1379	18	S	S	1	0
COG1415	18	S	S	1	0
COG1416	18	S	S	1	0
COG1417	18	S	S	1	0
COG1421	18	S	S	1	0
COG1430	18	S	S	1	0
COG1432	18	S	S	1	0
COG1433	18	S	S	1	0
COG1434	18	S	S	1	0
COG1458	18	S	S	1	0

COG1469	18	S	S	1	0
COG1478	18	S	S	1	0
COG1479	18	S	S	1	0
COG1496	18	S	S	1	0
COG1504	18	S	S	1	0
COG1507	18	S	S	1	0
COG1517	18	S	S	1	0
COG1518	18	S	S	1	0
COG1542	18	S	S	1	0
COG1543	18	S	S	1	0
COG1556	18	S	S	1	0
COG1565	18	S	S	1	0
COG1567	18	S	S	1	0
COG1572	18	S	S	1	0
COG1576	18	S	S	1	0
COG1578	18	S	S	1	0
COG1583	18	S	S	1	0
COG1590	18	S	S	1	0
COG1598	18	S	S	1	0
COG1602	18	S	S	1	0
COG1604	18	S	S	1	0
COG1610	18	S	S	1	0
COG1615	18	S	S	1	0
COG1617	18	S	S	1	0
COG1624	18	S	S	1	0
COG1628	18	S	S	1	0
COG1633	18	S	S	1	0
COG1641	18	S	S	1	0
COG1656	18	S	S	1	0
COG1679	18	S	S	1	0
COG1683	18	S	S	1	0
COG1688	18	S	S	1	0
COG1690	18	S	S	1	0
COG1700	18	S	S	1	0
COG1704	18	S	S	1	0
COG1720	18	S	S	1	0
COG1721	18	S	S	1	0
COG1723	18	S	S	1	0
COG1738	18	S	S	1	0
COG1739	18	S	S	1	0
COG1751	18	S	S	1	0
COG1753	18	S	S	1	0
COG1756	18	S	S	1	0
COG1769	18	S	S	1	0
COG1786	18	S	S	1	0

COG1801	18	S	S	1	0
COG1809	18	S	S	1	0
COG1812	18	S	S	1	0
COG1833	18	S	S	1	0
COG1839	18	S	S	1	0
COG1851	18	S	S	1	0
COG1857	18	S	S	1	0
COG1865	18	S	S	1	0
COG1872	18	S	S	1	0
COG1873	18	S	S	1	0
COG1895	18	S	S	1	0
COG1900	18	S	S	1	0
COG1901	18	S	S	1	0
COG1906	18	S	S	1	0
COG1912	18	S	S	1	0
COG1915	18	S	S	1	0
COG1920	18	S	S	1	0
COG1935	18	S	S	1	0
COG1944	18	S	S	1	0
COG1945	18	S	S	1	0
COG1980	18	S	S	1	0
COG1986	18	S	S	1	0
COG1990	18	S	S	1	0
COG1991	18	S	S	1	0
COG1992	18	S	S	1	0
COG1993	18	S	S	1	0
COG2006	18	S	S	1	0
COG2013	18	S	S	1	0
COG2014	18	S	S	1	0
COG2026	18	S	S	1	0
COG2028	18	S	S	1	0
COG2029	18	S	S	1	0
COG2042	18	S	S	1	0
COG2078	18	S	S	1	0
COG2096	18	S	S	1	0
COG2105	18	S	S	1	0
COG2106	18	S	S	1	0
COG2127	18	S	S	1	0
COG2128	18	S	S	1	0
COG2135	18	S	S	1	0
COG2138	18	S	S	1	0
COG2161	18	S	S	1	0
COG2164	18	S	S	1	0
COG2210	18	S	S	1	0
COG2250	18	S	S	1	0

COG2253	18 S	S	1	0
COG2306	18 S	S	1	0
COG2320	18 S	S	1	0
COG2327	18 S	S	1	0
COG2361	18 S	S	1	0
COG2383	18 S	S	1	0
COG2403	18 S	S	1	0
COG2406	18 S	S	1	0
COG2410	18 S	S	1	0
COG2411	18 S	S	1	0
COG2412	18 S	S	1	0
COG2419	18 S	S	1	0
COG2425	18 S	S	1	0
COG2427	18 S	S	1	0
COG2428	18 S	S	1	0
COG2429	18 S	S	1	0
COG2430	18 S	S	1	0
COG2433	18 S	S	1	0
COG2436	18 S	S	1	0
COG2442	18 S	S	1	0
COG2445	18 S	S	1	0
COG2450	18 S	S	1	0
COG2454	18 S	S	1	0
COG2456	18 S	S	1	0
COG2457	18 S	S	1	0
COG2461	18 S	S	1	0
COG2469	18 S	S	1	0
COG2501	18 S	S	1	0
COG2606	18 S	S	1	0
COG2848	18 S	S	1	0
COG2850	18 S	S	1	0
COG2912	18 S	S	1	0
COG2921	18 S	S	1	0
COG2930	18 S	S	1	0
COG2938	18 S	S	1	0
COG2983	18 S	S	1	0
COG3013	18 S	S	1	0
COG3025	18 S	S	1	0
COG3111	18 S	S	1	0
COG3148	18 S	S	1	0
COG3163	18 S	S	1	0
COG3199	18 S	S	1	0
COG3246	18 S	S	1	0
COG3253	18 S	S	1	0
COG3254	18 S	S	1	0

COG3268	18	S	S	1	0
COG3270	18	S	S	1	0
COG3272	18	S	S	1	0
COG3273	18	S	S	1	0
COG3287	18	S	S	1	0
COG3294	18	S	S	1	0
COG3332	18	S	S	1	0
COG3339	18	S	S	1	0
COG3342	18	S	S	1	0
COG3346	18	S	S	1	0
COG3347	18	S	S	1	0
COG3349	18	S	S	1	0
COG3350	18	S	S	1	0
COG3358	18	S	S	1	0
COG3360	18	S	S	1	0
COG3361	18	S	S	1	0
COG3367	18	S	S	1	0
COG3375	18	S	S	1	0
COG3377	18	S	S	1	0
COG3379	18	S	S	1	0
COG3382	18	S	S	1	0
COG3384	18	S	S	1	0
COG3387	18	S	S	1	0
COG3391	18	S	S	1	0
COG3402	18	S	S	1	0
COG3403	18	S	S	1	0
COG3406	18	S	S	1	0
COG3410	18	S	S	1	0
COG3427	18	S	S	1	0
COG3439	18	S	S	1	0
COG3461	18	S	S	1	0
COG3465	18	S	S	1	0
COG3496	18	S	S	1	0
COG3506	18	S	S	1	0
COG3535	18	S	S	1	0
COG3538	18	S	S	1	0
COG3542	18	S	S	1	0
COG3543	18	S	S	1	0
COG3603	18	S	S	1	0
COG3673	18	S	S	1	0
COG3679	18	S	S	1	0
COG3681	18	S	S	1	0
COG3737	18	S	S	1	0
COG3760	18	S	S	1	0
COG3777	18	S	S	1	0

COG3785	18 S	S	1	0
COG3791	18 S	S	1	0
COG3831	18 S	S	1	0
COG0799	18 S	S	1	0
COG3804	18 S	S	1	0
COG2110	18 S	S	1	0
COG0401	18 S	S	1	0
COG1310	18 S	S	1	0
COG1774	18 S	S	1	0
COG1916	18 S	S	1	0
COG1968	18 S	S	1	0
COG1856	18 S	S	1	0
COG1791	18 S	S	1	0
COG1917	18 S	S	1	0
COG3837	18 S	S	1	0
COG0599	18 S	S	1	0
COG3042	18 S	S	1	0
COG2886	18 S	S	1	0
COG1255	18 S	S	1	0
COG1303	18 S	S	1	0
COG1356	18 S	S	1	0
COG1460	18 S	S	1	0
COG1531	18 S	S	1	0
COG1627	18 S	S	1	0
COG1630	18 S	S	1	0
COG1650	18 S	S	1	0
COG1665	18 S	S	1	0
COG1667	18 S	S	1	0
COG1673	18 S	S	1	0
COG1689	18 S	S	1	0
COG1693	18 S	S	1	0
COG1698	18 S	S	1	0
COG1701	18 S	S	1	0
COG1710	18 S	S	1	0
COG1711	18 S	S	1	0
COG1771	18 S	S	1	0
COG1772	18 S	S	1	0
COG1790	18 S	S	1	0
COG1810	18 S	S	1	0
COG1817	18 S	S	1	0
COG1844	18 S	S	1	0
COG1849	18 S	S	1	0
COG1852	18 S	S	1	0
COG1860	18 S	S	1	0
COG1885	18 S	S	1	0

COG1888	18 S	S	1	0
COG1891	18 S	S	1	0
COG1892	18 S	S	1	0
COG1909	18 S	S	1	0
COG1931	18 S	S	1	0
COG2043	18 S	S	1	0
COG2083	18 S	S	1	0
COG2090	18 S	S	1	0
COG2098	18 S	S	1	0
COG2122	18 S	S	1	0
COG2880	18 S	S	1	0
COG2881	18 S	S	1	0
COG2892	18 S	S	1	0
COG3269	18 S	S	1	0
COG3286	18 S	S	1	0
COG3363	18 S	S	1	0
COG3365	18 S	S	1	0
COG3366	18 S	S	1	0
COG3369	18 S	S	1	0
COG3370	18 S	S	1	0
COG3372	18 S	S	1	0
COG3373	18 S	S	1	0
COG3388	18 S	S	1	0
COG3389	18 S	S	1	0
COG3390	18 S	S	1	0
COG3398	18 S	S	1	0
COG3430	18 S	S	1	0
COG3612	18 S	S	1	0
COG1745	18 S	S	1	0
COG0718	18 S	S	1	0
COG0779	18 S	S	1	0
COG1262	18 S	S	1	0
COG1284	18 S	S	1	0
COG1302	18 S	S	1	0
COG1307	18 S	S	1	0
COG1315	18 S	S	1	0
COG1322	18 S	S	1	0
COG1376	18 S	S	1	0
COG1385	18 S	S	1	0
COG1426	18 S	S	1	0
COG1481	18 S	S	1	0
COG1547	18 S	S	1	0
COG1550	18 S	S	1	0
COG1636	18 S	S	1	0
COG1649	18 S	S	1	0

COG1655	18	S	S	1	0
COG1664	18	S	S	1	0
COG1666	18	S	S	1	0
COG1671	18	S	S	1	0
COG1692	18	S	S	1	0
COG1699	18	S	S	1	0
COG1728	18	S	S	1	0
COG1729	18	S	S	1	0
COG1742	18	S	S	1	0
COG1755	18	S	S	1	0
COG1799	18	S	S	1	0
COG1806	18	S	S	1	0
COG1934	18	S	S	1	0
COG1937	18	S	S	1	0
COG1939	18	S	S	1	0
COG1963	18	S	S	1	0
COG1978	18	S	S	1	0
COG2001	18	S	S	1	0
COG2052	18	S	S	1	0
COG2121	18	S	S	1	0
COG2155	18	S	S	1	0
COG2170	18	S	S	1	0
COG2187	18	S	S	1	0
COG2258	18	S	S	1	0
COG2268	18	S	S	1	0
COG2307	18	S	S	1	0
COG2308	18	S	S	1	0
COG2315	18	S	S	1	0
COG2318	18	S	S	1	0
COG2326	18	S	S	1	0
COG2331	18	S	S	1	0
COG2343	18	S	S	1	0
COG2350	18	S	S	1	0
COG2353	18	S	S	1	0
COG2354	18	S	S	1	0
COG2357	18	S	S	1	0
COG2359	18	S	S	1	0
COG2377	18	S	S	1	0
COG2380	18	S	S	1	0
COG2604	18	S	S	1	0
COG2718	18	S	S	1	0
COG2719	18	S	S	1	0
COG2731	18	S	S	1	0
COG2739	18	S	S	1	0
COG2764	18	S	S	1	0

COG2815	18 S	S	1	0
COG2828	18 S	S	1	0
COG2830	18 S	S	1	0
COG2832	18 S	S	1	0
COG2833	18 S	S	1	0
COG2835	18 S	S	1	0
COG2836	18 S	S	1	0
COG2839	18 S	S	1	0
COG2840	18 S	S	1	0
COG2841	18 S	S	1	0
COG2845	18 S	S	1	0
COG2847	18 S	S	1	0
COG2849	18 S	S	1	0
COG2852	18 S	S	1	0
COG2859	18 S	S	1	0
COG2861	18 S	S	1	0
COG2898	18 S	S	1	0
COG2899	18 S	S	1	0
COG2900	18 S	S	1	0
COG2904	18 S	S	1	0
COG2908	18 S	S	1	0
COG2911	18 S	S	1	0
COG2914	18 S	S	1	0
COG2922	18 S	S	1	0
COG2926	18 S	S	1	0
COG2928	18 S	S	1	0
COG2929	18 S	S	1	0
COG2947	18 S	S	1	0
COG2952	18 S	S	1	0
COG2954	18 S	S	1	0
COG2958	18 S	S	1	0
COG2960	18 S	S	1	0
COG2964	18 S	S	1	0
COG2966	18 S	S	1	0
COG2968	18 S	S	1	0
COG2975	18 S	S	1	0
COG2976	18 S	S	1	0
COG2979	18 S	S	1	0
COG2984	18 S	S	1	0
COG2989	18 S	S	1	0
COG2990	18 S	S	1	0
COG2991	18 S	S	1	0
COG2996	18 S	S	1	0
COG3002	18 S	S	1	0
COG3009	18 S	S	1	0

COG3011	18 S	S	1	0
COG3012	18 S	S	1	0
COG3014	18 S	S	1	0
COG3018	18 S	S	1	0
COG3021	18 S	S	1	0
COG3022	18 S	S	1	0
COG3024	18 S	S	1	0
COG3027	18 S	S	1	0
COG3028	18 S	S	1	0
COG3034	18 S	S	1	0
COG3035	18 S	S	1	0
COG3036	18 S	S	1	0
COG3037	18 S	S	1	0
COG3041	18 S	S	1	0
COG3045	18 S	S	1	0
COG3055	18 S	S	1	0
COG3068	18 S	S	1	0
COG3074	18 S	S	1	0
COG3076	18 S	S	1	0
COG3078	18 S	S	1	0
COG3079	18 S	S	1	0
COG3082	18 S	S	1	0
COG3084	18 S	S	1	0
COG3085	18 S	S	1	0
COG3089	18 S	S	1	0
COG3090	18 S	S	1	0
COG3091	18 S	S	1	0
COG3092	18 S	S	1	0
COG3094	18 S	S	1	0
COG3097	18 S	S	1	0
COG3098	18 S	S	1	0
COG3099	18 S	S	1	0
COG3100	18 S	S	1	0
COG3101	18 S	S	1	0
COG3102	18 S	S	1	0
COG3105	18 S	S	1	0
COG3108	18 S	S	1	0
COG3110	18 S	S	1	0
COG3112	18 S	S	1	0
COG3117	18 S	S	1	0
COG3120	18 S	S	1	0
COG3122	18 S	S	1	0
COG3123	18 S	S	1	0
COG3124	18 S	S	1	0
COG3126	18 S	S	1	0

COG3132	18 S	S	1	0
COG3139	18 S	S	1	0
COG3140	18 S	S	1	0
COG3141	18 S	S	1	0
COG3146	18 S	S	1	0
COG3147	18 S	S	1	0
COG3151	18 S	S	1	0
COG3159	18 S	S	1	0
COG3165	18 S	S	1	0
COG3169	18 S	S	1	0
COG3171	18 S	S	1	0
COG3177	18 S	S	1	0
COG3181	18 S	S	1	0
COG3184	18 S	S	1	0
COG3189	18 S	S	1	0
COG3195	18 S	S	1	0
COG3196	18 S	S	1	0
COG3198	18 S	S	1	0
COG3204	18 S	S	1	0
COG3214	18 S	S	1	0
COG3216	18 S	S	1	0
COG3218	18 S	S	1	0
COG3219	18 S	S	1	0
COG3220	18 S	S	1	0
COG3222	18 S	S	1	0
COG3224	18 S	S	1	0
COG3225	18 S	S	1	0
COG3226	18 S	S	1	0
COG3228	18 S	S	1	0
COG3234	18 S	S	1	0
COG3236	18 S	S	1	0
COG3237	18 S	S	1	0
COG3238	18 S	S	1	0
COG3242	18 S	S	1	0
COG3247	18 S	S	1	0
COG3249	18 S	S	1	0
COG3251	18 S	S	1	0
COG3263	18 S	S	1	0
COG3266	18 S	S	1	0
COG3274	18 S	S	1	0
COG3295	18 S	S	1	0
COG3296	18 S	S	1	0
COG3310	18 S	S	1	0
COG3314	18 S	S	1	0
COG3323	18 S	S	1	0

COG3330	18 S	S	1	0
COG3333	18 S	S	1	0
COG3334	18 S	S	1	0
COG3337	18 S	S	1	0
COG3394	18 S	S	1	0
COG3395	18 S	S	1	0
COG3396	18 S	S	1	0
COG3397	18 S	S	1	0
COG3399	18 S	S	1	0
COG3400	18 S	S	1	0
COG3412	18 S	S	1	0
COG3416	18 S	S	1	0
COG3421	18 S	S	1	0
COG3422	18 S	S	1	0
COG3453	18 S	S	1	0
COG3455	18 S	S	1	0
COG3472	18 S	S	1	0
COG3482	18 S	S	1	0
COG3490	18 S	S	1	0
COG3492	18 S	S	1	0
COG3494	18 S	S	1	0
COG3495	18 S	S	1	0
COG3501	18 S	S	1	0
COG3502	18 S	S	1	0
COG3512	18 S	S	1	0
COG3513	18 S	S	1	0
COG3514	18 S	S	1	0
COG3515	18 S	S	1	0
COG3516	18 S	S	1	0
COG3517	18 S	S	1	0
COG3518	18 S	S	1	0
COG3519	18 S	S	1	0
COG3520	18 S	S	1	0
COG3521	18 S	S	1	0
COG3522	18 S	S	1	0
COG3523	18 S	S	1	0
COG3528	18 S	S	1	0
COG3530	18 S	S	1	0
COG3533	18 S	S	1	0
COG3536	18 S	S	1	0
COG3544	18 S	S	1	0
COG3551	18 S	S	1	0
COG3553	18 S	S	1	0
COG3554	18 S	S	1	0
COG3558	18 S	S	1	0

COG3564	18	S	S	1	0
COG3566	18	S	S	1	0
COG3567	18	S	S	1	0
COG3575	18	S	S	1	0
COG3580	18	S	S	1	0
COG3581	18	S	S	1	0
COG3584	18	S	S	1	0
COG3586	18	S	S	1	0
COG3589	18	S	S	1	0
COG3592	18	S	S	1	0
COG3595	18	S	S	1	0
COG3602	18	S	S	1	0
COG3610	18	S	S	1	0
COG3644	18	S	S	1	0
COG3649	18	S	S	1	0
COG3651	18	S	S	1	0
COG3657	18	S	S	1	0
COG3662	18	S	S	1	0
COG3665	18	S	S	1	0
COG3680	18	S	S	1	0
COG3685	18	S	S	1	0
COG3691	18	S	S	1	0
COG3692	18	S	S	1	0
COG3708	18	S	S	1	0
COG3735	18	S	S	1	0
COG3738	18	S	S	1	0
COG3742	18	S	S	1	0
COG3743	18	S	S	1	0
COG3744	18	S	S	1	0
COG3749	18	S	S	1	0
COG3750	18	S	S	1	0
COG3753	18	S	S	1	0
COG3755	18	S	S	1	0
COG3756	18	S	S	1	0
COG3758	18	S	S	1	0
COG3763	18	S	S	1	0
COG3778	18	S	S	1	0
COG3779	18	S	S	1	0
COG3782	18	S	S	1	0
COG3784	18	S	S	1	0
COG3786	18	S	S	1	0
COG3787	18	S	S	1	0
COG3789	18	S	S	1	0
COG3792	18	S	S	1	0
COG3795	18	S	S	1	0

COG3796	18	S	S	1	0
COG3797	18	S	S	1	0
COG3798	18	S	S	1	0
COG3801	18	S	S	1	0
COG3802	18	S	S	1	0
COG3803	18	S	S	1	0
COG3807	18	S	S	1	0
COG3809	18	S	S	1	0
COG3811	18	S	S	1	0
COG3812	18	S	S	1	0
COG3813	18	S	S	1	0
COG3814	18	S	S	1	0
COG3816	18	S	S	1	0
COG3820	18	S	S	1	0
COG3822	18	S	S	1	0
COG3824	18	S	S	1	0
COG3825	18	S	S	1	0
COG3826	18	S	S	1	0
COG3827	18	S	S	1	0
COG3828	18	S	S	1	0
COG3832	18	S	S	1	0
COG3309	18	S	S	1	0
COG2257	18	S	S	1	0
COG1747	18	S	S	1	0
COG2302	18	S	S	1	0
COG3739	18	S	S	1	0
COG3790	18	S	S	1	0
COG3176	18	S	S	1	0
COG3429	18	S	S	1	0
COG2720	18	S	S	1	0
COG1326	18	S	S	1	0
COG1340	18	S	S	1	0
COG1795	18	S	S	1	0
COG2034	18	S	S	1	0
COG2959	18	S	S	1	0
COG3299	18	S	S	1	0
COG3182	18	S	S	1	0
COG3016	18	S	S	1	0
COG3128	18	S	S	1	0
COG3767	18	S	S	1	0
COG1357	18	S	S	1	0
COG0700	18	S	S	1	0
COG1238	18	S	S	1	0
COG1285	18	S	S	1	0
COG1288	18	S	S	1	0

COG1289	18 S	S	1	0
COG1297	18 S	S	1	0
COG1300	18 S	S	1	0
COG1422	18 S	S	1	0
COG2245	18 S	S	1	0
COG2246	18 S	S	1	0
COG2339	18 S	S	1	0
COG2426	18 S	S	1	0
COG2431	18 S	S	1	0
COG2707	18 S	S	1	0
COG2717	18 S	S	1	0
COG2733	18 S	S	1	0
COG2855	18 S	S	1	0
COG2862	18 S	S	1	0
COG2949	18 S	S	1	0
COG3059	18 S	S	1	0
COG3127	18 S	S	1	0
COG3134	18 S	S	1	0
COG3152	18 S	S	1	0
COG3162	18 S	S	1	0
COG3164	18 S	S	1	0
COG3174	18 S	S	1	0
COG3205	18 S	S	1	0
COG3212	18 S	S	1	0
COG3223	18 S	S	1	0
COG3229	18 S	S	1	0
COG3235	18 S	S	1	0
COG3304	18 S	S	1	0
COG3305	18 S	S	1	0
COG3308	18 S	S	1	0
COG3463	18 S	S	1	0
COG3601	18 S	S	1	0
COG3619	18 S	S	1	0
COG3686	18 S	S	1	0
COG3714	18 S	S	1	0
COG3748	18 S	S	1	0
COG3752	18 S	S	1	0
COG3821	18 S	S	1	0
COG1714	18 S	S	1	0
COG1814	18 S	S	1	0
COG0586	18 S	S	1	0
COG2512	18 S	S	1	0
COG2389	18 S	S	1	0
COG2995	18 S	S	1	0
COG3007	18 S	S	1	0

COG3678	18	S	S	1	0
COG3747	18	S	S	1	0
COG3600	18	S	S	1	0
COG3645	18	S	S	1	0
COG3646	18	S	S	1	0
COG1652	18	S	S	1	0
COG2348	18	S	S	1	0
COG2340	18	S	S	1	0
COG2369	18	S	S	1	0
COG1659	18	S	S	1	0
COG3257	18	S	S	1	0
COG3197	18	S	S	1	0
COG3615	18	S	S	1	0
COG3193	18	S	S	1	0
COG3597	18	S	S	1	0
COG1520	18	S	S	1	0
COG2120	18	S	S	1	0
COG1619	18	S	S	1	0
COG3155	18	S	S	1	0
COG2363	18	S	S	1	0
COG2879	18	S	S	1	0
COG1561	18	S	S	1	0
				3305	2
				0.999395222	0.00060478
